# Roles of Focal Adhesion Kinase PTK2 and Integrin αIIbβ3 Signaling in Collagen- and GPVI-Dependent Thrombus Formation under Shear

**DOI:** 10.3390/ijms23158688

**Published:** 2022-08-04

**Authors:** Jingnan Huang, Natalie J. Jooss, Delia I. Fernández, Albert Sickmann, Ángel García, Kanin Wichapong, Ingrid Dijkgraaf, Johan W. M. Heemskerk

**Affiliations:** 1 Department of Biochemistry, CARIM, Maastricht University, 6229 ER Maastricht, The Netherlands; 2 Leibniz Institut für Analytische Wissenschaften-ISAS-e.V., 44139 Dortmund, Germany; 3 Center for Research in Molecular Medicine and Chronic Diseases (CIMUS), Universidade de Santiago de Compostela and Instituto de Investigación Sanitaria de Santiago (IDIS), Barcelona Ave., 15782 Santiago de Compostela, Spain; 4 Institute of Cardiovascular Sciences, College of Medical and Dental Sciences, University of Birmingham, Birmingham B15 2TT, UK; 5 Medizinische Proteom-Center, Medizinische Fakultät, Ruhr-Universität Bochum, 44801 Bochum, Germany; 6 Department of Chemistry, College of Physical Sciences, University of Aberdeen, Aberdeen AB24 3FX, UK; 7 Synapse Research Institute Maastricht, Kon. Emmaplein 7, 6217 KD Maastricht, The Netherlands

**Keywords:** focal adhesion kinase, integrins, GPR56, platelets, thrombus formation

## Abstract

Glycoprotein (GP)VI and integrin αIIbβ3 are key signaling receptors in collagen-dependent platelet aggregation and in arterial thrombus formation under shear. The multiple downstream signaling pathways are still poorly understood. Here, we focused on disclosing the integrin-dependent roles of focal adhesion kinase (protein tyrosine kinase 2, PTK2), the shear-dependent collagen receptor GPR56 (*ADGRG1* gene), and calcium and integrin-binding protein 1 (CIB1). We designed and synthetized peptides that interfered with integrin αIIb binding (pCIB and pCIB^m^) or mimicked the activation of GPR56 (pGRP). The results show that the combination of pGRP with PTK2 inhibition or of pGRP with pCIB > pCIB^m^ in additive ways suppressed collagen- and GPVI-dependent platelet activation, thrombus buildup, and contraction. Microscopic thrombus formation was assessed by eight parameters (with script descriptions enclosed). The suppressive rather than activating effects of pGRP were confined to blood flow at a high shear rate. Blockage of PTK2 or interference of CIB1 no more than slightly affected thrombus formation at a low shear rate. Peptides did not influence GPVI-induced aggregation and Ca^2+^ signaling in the absence of shear. Together, these data reveal a shear-dependent signaling axis of PTK2, integrin αIIbβ3, and CIB1 in collagen- and GPVI-dependent thrombus formation, which is modulated by GPR56 and exclusively at high shear. This work thereby supports the role of PTK2 in integrin αIIbβ3 activation and signaling.

## 1. Introduction

Collagen- and fibrin(ogen)-induced platelet activation and aggregation through glycoprotein VI (GPVI) have been recognized as controlling processes of in vitro and in vivo arterial thrombus formation, with a limited role in hemostasis [1,2,3]. GPVI is an immunoglobin receptor that via the FcR γ-chain co-receptor, when triggered upon ligand binding, causes activation of a signal transduction pathway of protein tyrosine kinases, phosphatidylinositol-3-kinases, and phospholipase Cγ2 (PLCγ2) to evoke granule release and activation of integrin αIIbβ3 [4,5,6]. Fibrinogen is the major ligand for integrin αIIbβ3 to establish platelet aggregation and thrombus formation, in which the integrin can act in a non-redundant way with GPVI [7,8,9].

Recent multiparameter microfluidics assays have provided first understanding of the molecular mechanisms, implicated in whole-blood thrombus formation involving GPVI [10,11,12]. The heterogenous buildup of arterial thrombi takes place by a series of consecutive platelet activation events, i.e., flow-dependent platelet adhesion, integrin αIIbβ3 activation, granular secretion, platelet aggregation, and thrombus contraction and stabilization [8,13]. Mouse studies have further shown that several hundreds of platelet-expressed genes and proteins contribute to collagen-dependent arterial thrombus formation, thus pointing to a high complexity of the underlying platelet signaling pathways [14].

Knowing that multiple shear-dependent platelet adhesion mechanisms [15] and platelet-platelet interactions [11] can determine the thrombus-forming process, we used our experience in the targeted design and synthesis of anti-platelet peptides [13,15] to reveal the importance of less understood signaling pathways. In this context, a less well-studied protein acting downstream of αIIbβ3 in platelets is protein tyrosine kinase 2 (PTK2, also known as focal adhesion kinase) [16,17,18]. In various cell types, including platelets, PTK2 is known to accumulate in focal adhesion sites formed upon stretching forces [19,20]. In the mechanical force-dependent activation and signaling of integrins, PTK2 was found to regulate the adhesion-dependent talin activity and actomyosin dynamics [21]. Interestingly, PTK2 has recently become a potential therapeutic target for some cancers [22,23].

Both peptide inhibition and mouse knockout studies have shown that platelet PTK2 can become activated upon fibrinogen binding via the calcium and integrin-binding protein 1 (CIB1, gene CIB1). Interaction of CIB1 with the αIIb chain appeared to regulate the integrin outside-in signaling [24,25,26,27]. Proteomic analyses have revealed that PTK2 and CIB1 are expressed at reasonable numbers of 2,956 and 780 copies/platelet, respectively [28,29]. Yet, there is confusion if the role of CIB1 in platelets is stimulatory [30] or inhibitory [31]. Of note, the CIB1-PTK2 interaction occurs independently of the tyrosine kinase Syk, which binds to the other β3 integrin chain. In the absence of CIB1, platelet spreading on fibrinogen was found to be greatly impaired [24,26]. Evidence for this role of CIB1 came in part from the use of a αIIb cytoplasmic peptide (pCIB), entering platelets and interfering with the binding of CIB1 to αIIb; the peptide efficiently inhibited the human platelet spreading via the outside-in signaling mechanism [25]. Mouse knockout studies further proved that CIB1 contributes to arterial thrombosis and hemostasis [30].

A novel shear force-dependent receptor on platelets is the G protein-coupled receptor 56 (GPR56, gene *ADGRG1*), signaling via G13α to allow platelet shape change [32]. In both human and mouse platelets, ADGRG1 mRNA is expressed at relatively high levels [29]. In other cells, GPR56 appeared as an adhesive receptor for collagen type-III [33]. In initial papers, identifying platelet GPR56 as a shear-dependent collagen receptor, it appeared to become self-activated via its tethered ligand, after which signaling responses through other collagen receptors such as GPVI were enforced [32,34]. Knock-out mouse studies pointed to a moderate role of GPR56 in arterial thrombosis and hemostasis [32]. However, the precise requirements for GPR56 to support human thrombus formation on vascular collagens remain unclear.

In the present paper, we used a panel of inhibitors and synthetized peptides to re-investigate the roles of PTK2, CIB1, and GPR56 in shear-dependent thrombus formation on a range of collagen surfaces with variable GPVI dependency.

## 2. Results

### 2.1. Limited Effect of the GPR56 Peptide on Collagen Induced Thrombus Formation under Shear

To investigate how the novel collagen receptor GPR56 contributes to platelet adhesion and thrombus formation, we treated blood from healthy donors with the synthetized peptide pGRP (TYFAVLM, 50 μg/mL), corresponding to the hidden tethered ligand of GPR56, and hence mimicking the shear-dependent activation of this receptor [32]. Perfusion of the blood was performed at an arterial wall-shear rate of 1600 s^−1^ [11,35]. For platelet interaction, microspots of collagen I, collagen III, and collagen IV were used with decreasing GPVI dependency. As before, brightfield and multicolor microscopic images taken after perfusion were analyzed for eight parameters (Table 1), i.e., platelet adhesion (P1), platelet aggregation (P2), thrombus morphology, multilayer and contraction scores (P3–5), platelet activation marker phosphatidylserine exposure (P6), P-selectin expression (P7), and integrin αIIbβ3 activation (P8). An extended description of the scripts in Fiji to measure the continuous parameters and to obtain the discontinuous scores is given in the Supplementary Materials.

**Figure 1:**
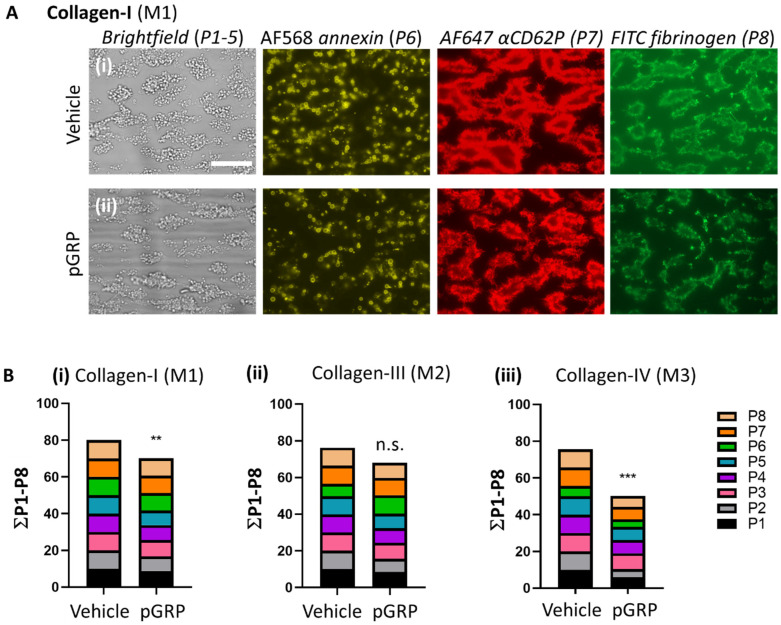
Effect of GPR56 interference on collagen-induced thrombus formation at a high shear rate. Whole blood (700 μL) was pre-incubated with vehicle medium or pGRP peptide (50 μg/mL) for 10 min. After recalcification, blood samples were perfused over microspots of collagen I, III, and IV for 3.5 min at wall-shear rate of 1600 s^−1^. Brightfield and fluorescence images were taken per microspot at end stage. (**A**) Shown are representative microscopic images for collagen I of (**i**) vehicle control runs or (**ii**) pGRP runs. Scale bar = 10 μm. Complementary images for thrombi on collagen III and collagen IV are shown in Figure S1. (**B**) Cumulative plots per condition of scaled (0–10) image parameters: P1, platelet adhesion; P2, platelet aggregate coverage; P3–5, thrombus morphology, multilayer and contraction scores; platelet activation markers: P6, PS exposure; P7, P-selectin expression; P8, fibrinogen binding (see Table 1). Shown are means of duplicate runs for three donors. Mean values ± SD (n = 3); n.s., not significant, ** *p* < 0.005, *** *p* < 0.001 vs. vehicle (paired Student’s *t*-test).

Representative images collected at end-stage for collagen I (Figure 1A) and for collagen III and IV (Figure S1A,B) indicated that treatment with pGRP did no more than slightly affect platelet adhesion and thrombus buildup (brightfield images) or platelet activation (3-color fluorescence images). Summative presentation of the univariate scaled parameter values pointed to an overall small inhibitory rather than stimulatory peptide effect, which was only significant for collagen I and IV (Figure 1(Bi–iii)). Similar results were obtained at the standard high shear rate of 1000 s^−1^. Control experiments did not point to a pGRP effect on collagen-induced platelet aggregation by conventional light transmission aggregometry (not shown, but see below).

### 2.2. Effects of the GPR56 Peptide Combined with PTK2 Inhibition on Thrombus Formation under Shear

**Figure 2:**
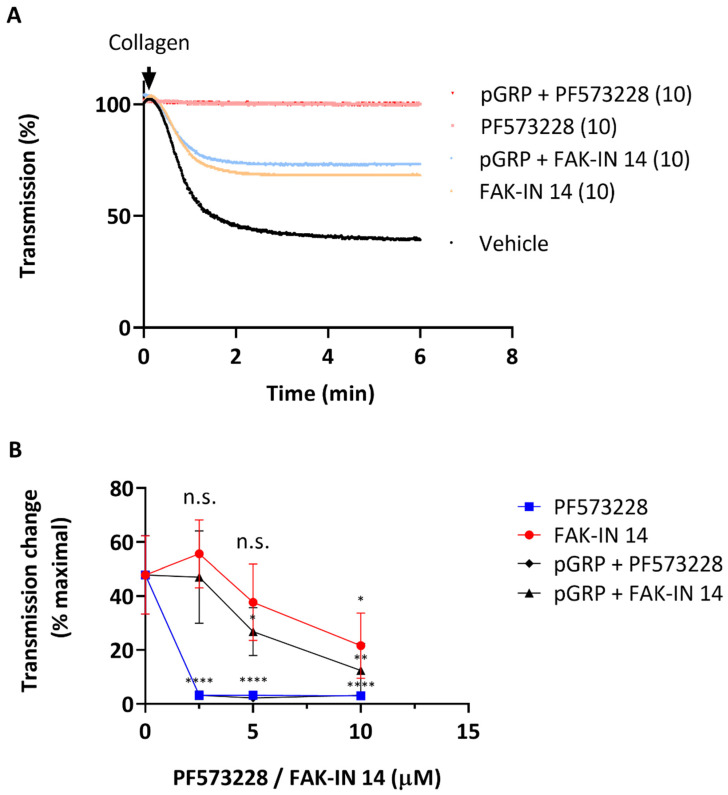
Effects of GPR56 and PTK2 interference on collagen-induced platelet aggregation. Washed platelets (250 × 10^9^/L) were incubated with vehicle (control), pGRP peptide (50 μg/mL), and indicated PTK2 inhibitor (2.5–10 μM) for 10 min. Platelet aggregation was monitored by light transmission in response to collagen I (1 μg/mL). (**A**) Representative traces of collagen-induced aggregation. (**B**) Dose-dependent effect of PTK2 inhibitors on maximal aggregation. Mean values ± SD (n = 3 donors); n.s., not significant, * *p* < 0.05, ** *p* < 0.005, **** *p* < 0.0001 vs. vehicle (paired Student’s *t*-test).

Given the proposed mechanism of GPR56 in shear- and G13α-dependent platelet activation [32], we hypothesized that its role could be masked by integrin αIIbβ3 outside-in signaling, in which the focal adhesion kinase PTK2 plays a central role [17,36,37]. To investigate this, we used two structurally different PTK2 inhibitors, PF573228 and FAK-IN14, of which the former was tested before on human platelets [38,39]. Initial dose-response experiments (2.5–10 μΜ) indicated a more potent effect of PF573228 than of FAK-IN14 on collagen-induced platelet aggregation (Figure 2A,B), which agrees with earlier published IC_50_ values [38,40]. As expected, under the slowly stirred conditions (1200 rpm providing a low shear rate) in light transmission aggregometry, the addition of pGRP did not further enhance the dose-dependent inhibitory effects of PF573228 or FAK-IN14 on platelet aggregation (Figure 2B).

**Figure 3:**
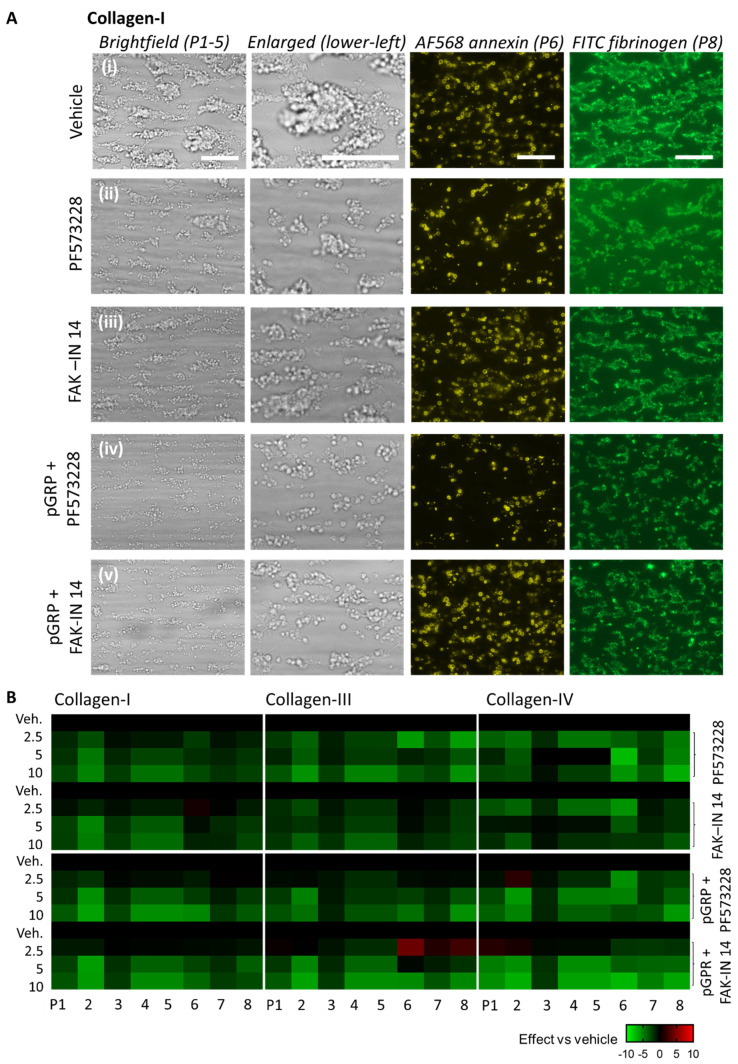
Effects of GPR56-binding peptide and PTK2 inhibition on collagen-induced thrombus formation. Whole blood samples were pre-incubated with vehicle medium (control) or indicated PTK2 inhibitor (PF573228 or FAK-IN14 at 2.5–10 μM) with or without pGRP peptide (50 μg/mL) for 10 min. After recalcification, the blood was perfused over collagen I, III, and IV for 3.5 min at a standard shear rate of 1000 s^−1^. End-stage brightfield and fluorescence images were analyzed for thrombus parameters P1–8. Enlarged images (lower-left corner) are indicated to visualize the formed platelet aggregates. (**A**) Representative images for collagen I of (**i**) vehicle control, (**ii**) PF573228 (10 μM), (**iii**) FAK-IN14 (10 μM), (**iv**) pGRP + PF573228 runs, and (**v**) pGRP + FAK-IN14. Scale bar = 10 μm. Representative images for collagen III and collagen IV are shown in Figure S2. (**B**) Subtraction heatmap representing control-subtracted scaled (0–10) parameter values for collagen I, III, and IV microspots. The color code represents a decrease (green) or increase (red) in comparison to control runs. Means of duplicate runs for three donors were compared per blood sample. For statistics, see Figure S3.

To investigate how PTK2 inhibition affected whole-blood thrombus formation, we used the same microfluidic setup with collagen I, III, and IV. Blood samples were pretreated with 2.5–10 μΜ of PF573228 or FAK-IN14 and then perfused over the collagen spots at the standard high shear rate of 1000 s^−1^. Inspection of microscopic images showed a consistent effect of PF573228 and FAK-IN14 at the highest dose applied by reducing platelet aggregate formation on collagen I (Figure 3Ai–iii). Subtraction heatmap analysis after parameter scaling showed that the reduction with either inhibitor extended to the majority of parameters, as well as from collagen I and III to collagen IV (Figure 3B). This was confirmed by summation of the univariate scaled parameters P1–8, pointing to a significant reduction for PF573228 > FAK-IN14 (Figure S2A–C, panel i).

We then examined the combined effect with the GPR56 peptide added. Surprisingly, the combination of pGRP with PF573228 or FAK-IN14 further reduced platelet adhesion and aggregate size on collagen I (Figure 3Aiv,v), as well as collagen III and IV (Figure S3ii,iii). In particular for PF573228, this reduction extended to a lower phosphatidylserine exposure, a marker of GPVI activity. This was also apparent from the subtraction heatmap of Figure 3B, showing larger effect sizes with the combined presence on all surfaces. Cumulative analysis of the scaled parameters confirmed that the reduction with pGRP was highest for PF573228 (Figure S2).

**Figure 4:**
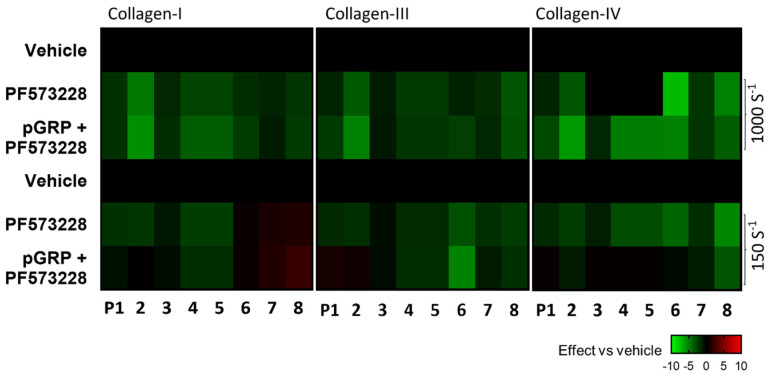
Shear rate dependency of GPR56 and PTK2 interference. Blood samples pre-incubated with vehicle (control) or PF573228 (5 μM) with/ without pGRP (50 μg/mL) for 10 min and then perfused over collagen microspots for 3.5 or 6 min at a wall-shear rate of 1000 s^−1^ or 150 s^−1^. Parameter analysis of recorded images was as for Figure 1. Shown is a subtraction heatmap representing control-subtracted scaled (0–10) parameter values for collagen I, III, and IV microspots. The color code represents a decrease (green) or increase (red) in comparison to vehicle control runs.

Since activation of the GPR56 receptor is considered to act in a shear-dependent way, we compared the effects of PF573228 (5 μM) alone or in combination with pGRP (50 μg/mL) at high (1000 s^−1^) and low (150 s^−1^) shear rates. The composed subtraction heatmap versus the vehicle control condition pointed to an essential lack of effect by the combination with pGRP at a low shear rate, in contrast to the high-shear condition (Figure 4). This also appeared from the cumulation of scaled parameter values (Figure S4). Taken together, these results pointed to a shear-dependent suppression of collagen-dependent thrombus by interfering with GPR56 plus PTK2 activity.

### 2.3. In Silico Design of CIB1-Interfering Peptide Binding to Integrin Chain αIIb

**Figure 5:**
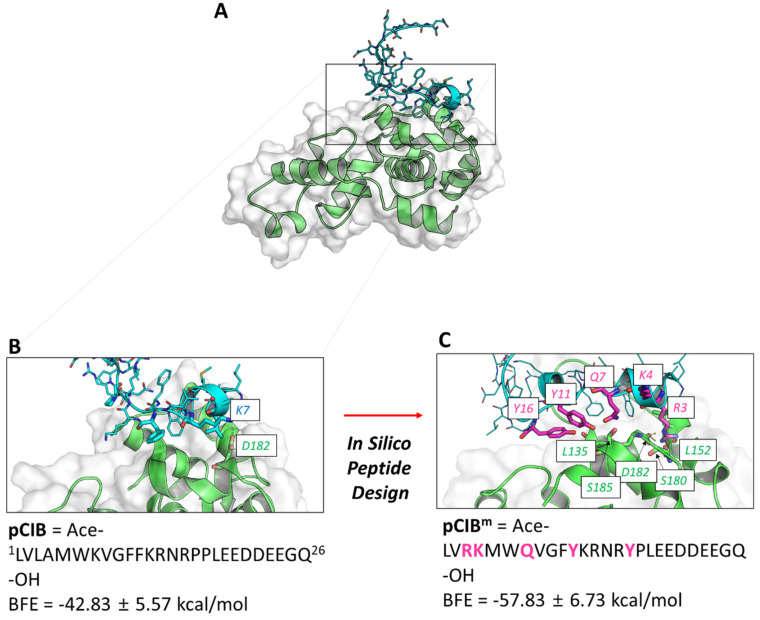
Molecular dynamics simulation of the pCIB–CIB1 complex formation. (**A**) Reported structure of CIB1 in complex with the pCIB peptide, mimicking part of the intracellular αIIb chain. (**B**) Calculated structure of the pCIB–CIB1 complex obtained by molecular dynamics simulation. (**C**) Structure of the modified pCIB^m^-CIB1 complex by molecular dynamics simulation. Color code: hydrogen bonds shown as yellow dashed lines; amino acid residues of the wildtype (**B**) and mutated (**C**) peptides are indicated in cyan and magenta, respectively; CIB1 residues are pictured in green; also indicated per peptide is the calculated binding free energy (BFE).

Since PTK2 is known to be activated via integrin αIIbβ3 outside-in signaling, involving binding of CIB1 to the αIIb chain, we used the αIIb cytoplasmic peptide pCIB, which has been shown to enter into platelets and block the CIB1-αIIb interaction, as evidenced by the suppression of platelet spreading on fibrinogen [24,26]. Virtual analysis of the binding mode of the 26 amino-acid pCIB to the binding pocket of CIB1 revealed a free binding energy of −43 kcal/mol (Figure 5A,B). Aiming to improve the predicted binding affinity of the peptide with CIB1, we rationally mutated several residues of pCIB in silico, which resulted in a list of 31 mutant peptides. Among these, the peptide pCIB^m^ with five mutations had the lowest binding free energy of −58 kcal/mol and predicted an unaltered interaction mode with CIB1 (Figure 5C). We then chemically synthetized the previously used pCIB and the pCIB^m^ peptide for microfluidic assays. After purification, the measured masses of pCIB (3104 Da) and pCIB^m^ (3286 Da) corresponded well with the theoretical monoisotopic masses of 3102 Da and 3284 Da, respectively.

### 2.4. Effects of Combined Peptides Interfering with GPR56 and CIB1 on Thrombus Formation under Shear

**Figure 6:**
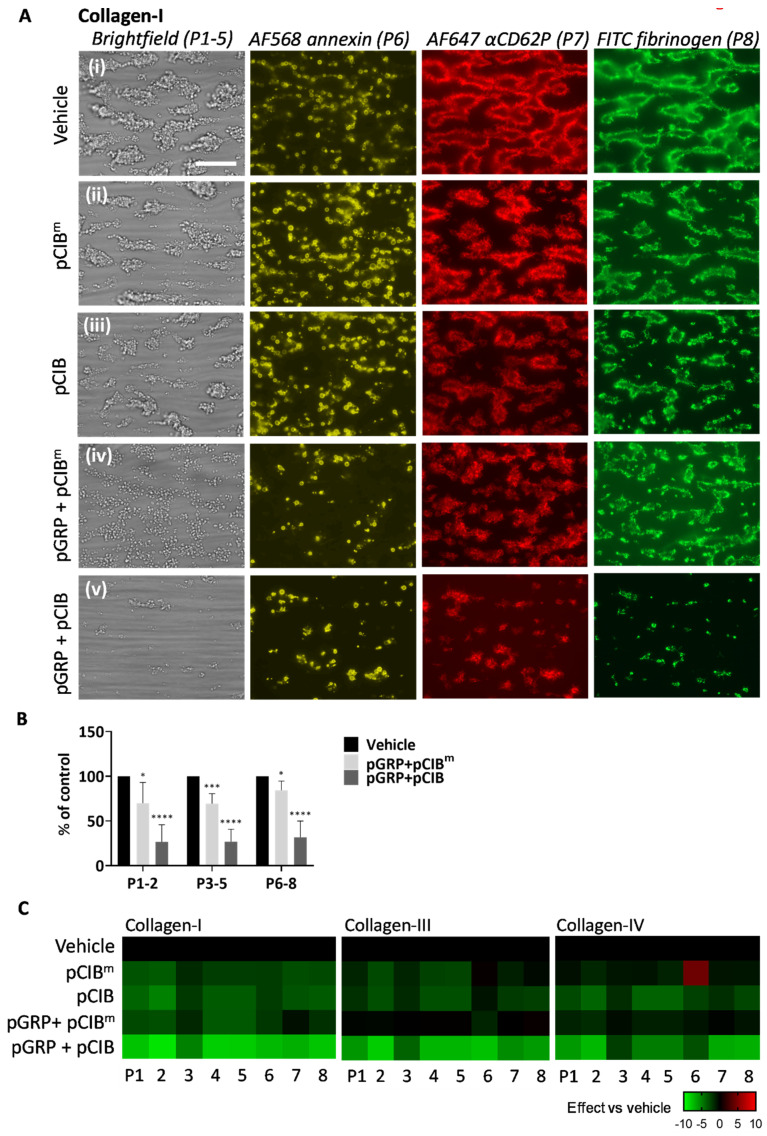
Combined GPR56 and CIB1 peptides affecting collagen-induced thrombus formation. Blood samples were pre-incubated with vehicle medium (control) or indicated peptides pGRP, pCIB, and pCIB^m^ (50 μg/mL each) for 10 min. Thrombus formation on collagen I, III, and IV was monitored. (**A**) Representative images for collagen I of (**i***)* vehicle control, (**ii**) pCIB^m^, (**iii**) pCIB, and (**iv**) pGRP + pCIB^m^, or (**v**) pGRP + pCIB. Scale bar = 10 μm. (**B**) Percentual effects of peptides on combined parameters of platelet deposition (P1–2), thrombus characteristics (P3–5), and platelet activation (P6–8) versus the vehicle control condition. Additional images and raw data for collagen III and collagen IV are given in Figures S5 and S6. (**C**) Subtraction heatmap representing control-subtracted scaled (0–10) parameter values for collagen I, III, and IV microspots. The color code represents a decrease (green) or increase (red) in comparison to controls. Mean values ± SD (n = 3 donors). * *p* < <0.05, *** *p* < 0.001, **** *p* < 0.0001 vs. vehicle (paired Student’s *t*-test).

**Figure 7:**
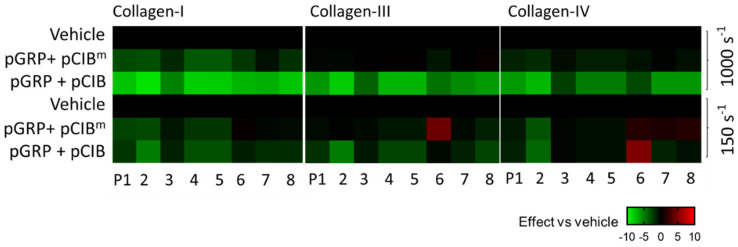
Shear rate dependency of GPR56 and CIB1 peptides. Blood samples pre-incubated with vehicle (control) or indicated peptides (50 μg/mL) for 10 min and then perfused over collagen microspots for 3.5 min at 1000 s^−1^ or for 6 min at 150 s^−1^. Shown is the subtraction heatmap representing control-subtracted scaled (0–10) image parameter values for collagen I, III, and IV microspots. The color code represents a decrease (green) or increase (red) in comparison to vehicle control runs.

For flow assays, the two synthetized CIB1-binding peptides were used at an affordable high dose for platelet-inhibiting peptides of 50 μg/mL in whole blood [15]. Preincubation of blood with the pCIB or pCIB^m^ resulted in no more than small visual effects on thrombus formation at microspots of collagen I (Figure 6Ai–iii). We also investigated the effects of combined application of pCIB or pCIB^m^ and pGRP. This combined treatment showed for collagen I a reduction in platelet adhesion, thrombus buildup, and platelet activation markers including exposed PS and P-selectin and activated integrin αIIbβ3, of which the reduction was more prominent for pCIB than for pCIB^m^ (Figure 6Aiv,v). The stronger effect with pCIB was confirmed by statistical analysis of the parameters of platelet deposition (P1–2), thrombus characteristics (P3–5), and platelet activation (P6–8) (Figure 6B). Regarding collagen III and IV, representative images showed similar effects of the combined addition of peptides (Figure S5A,B). Upon quantification, pGRP plus pCIB significantly reduced platelet deposition, thrombus characteristics, and platelet activation (Figure S6 Ai,ii). A synergistic effect of pGRP + pCIB (but not pCIB^m^) also appeared from the subtraction heatmap (Figure 7) and from cumulative plots of the scaled parameters (Figure S6Bi–iii). For all collagen types, effect sizes decreased in the order of pGRP + pCIB > pCIB > pGRP + pCIB^m^ > pCIB^m^.

To assess shear dependency of these effects, we again evaluated the effects on thrombus formation inhibition of pGRP with/without pCIB or pCIB^m^ at high (1000 s^−1^) and low (150 s^−1^) shear rates. The subtraction heatmap pointed to a consistently larger effect of the pGRP + pCIB combination, regardless of parameter and collagen type, at a high shear rate than at a low shear rate (Figure 7). This was confirmed by statistical parameter analysis (Figure S7).

### 2.5. Absence of Peptide Effects on GPVI-Induced Platelet Aggregation and Ca^2+^ Responses

**Figure 8:**
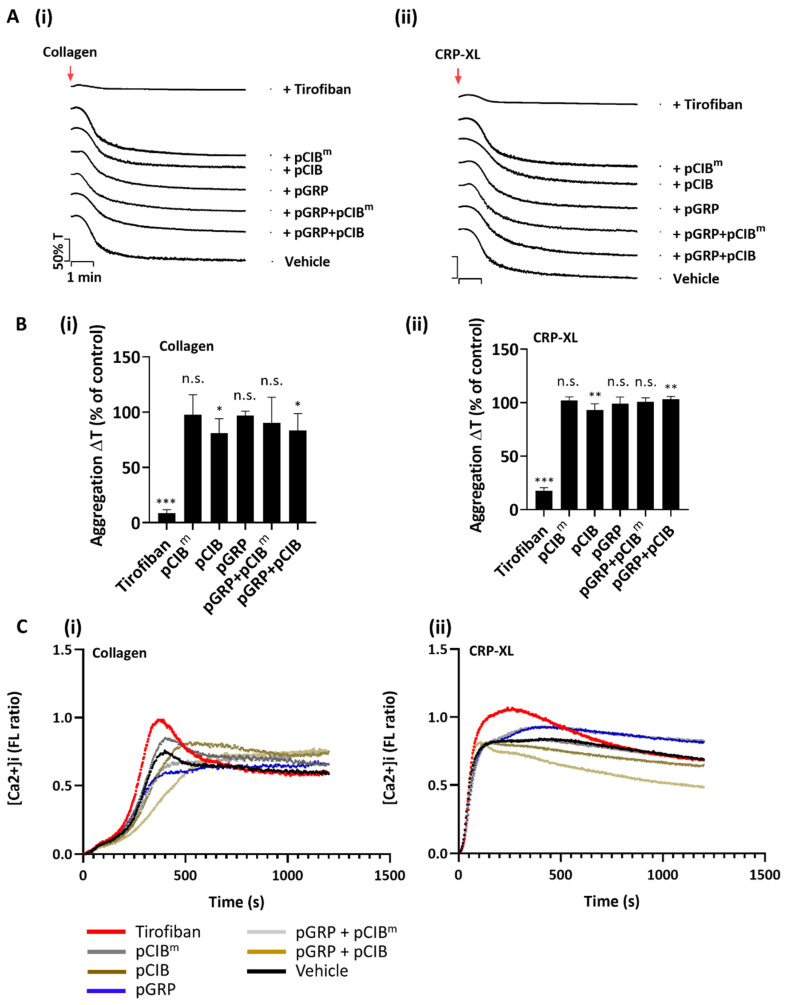
No effect of combined peptides pGRP and pCIB on collagen-induced platelet aggregation or Ca^2+^ fluxes. (**A**,**B**) Platelet preparations (250 × 10^8^/L) were pre-incubated with vehicle control, tirofiban (1 μg/mL) or indicated peptides (50 μg/mL) for 10 min. Platelet aggregation was monitored by light transmission aggregometry in response to 1 μg/mL collagen I or (**i**) or 1 μg/mL CRP-XL (**ii**). (**A**) representative aggregation traces (**B**) and normalized transmission changes. (**C**) Fura-2-loaded platelets were pre-incubated with vehicle control, tirofiban (1 μg/mL), or indicated peptides (50 μg/mL) for 10 min, before addition to 96-well plates. After supplementation of 1 mM CaCl_2_, loaded platelets were automatically stimulated with 10 μg/mL collagen I (**i**) or 10 μg/mL CRP-XL (**ii**). Dual wavelength 340/380 nm fluorescence changes per well were recorded in a FlexStation 3. Shown are representative [Ca^2+^]_i_ traces per agonist. Mean values ± SD (n = 3 donors); n.s., not significant, * *p* < 0.05, ** *p* < 0.005, *** *p* < 0.001, vs. vehicle (paired Student’s *t*-test).

Considering that the peptide-sensitive interaction of CIB1 with αIIbβ3 is thought to be confined to outside-in signaling [25], we also checked the effects of pGRP in combination with pCIB or pCIB^m^ on GPVI-mediated platelet aggregation and intracellular Ca^2+^ responses. As a control, we used the common αIIbβ3 inhibitor tirofiban [13]. As expected, up to a concentration of 50 μg/mL, the peptide combination was unable to suppress the platelet aggregation response to collagen I or CRP-XL (Figure 8Ai,ii). Quantitative analysis only showed a minor significant aggregation inhibition by pCIB and pGRP + pCIB (Figure 8B). The control compound tirofiban completely blocked collagen I- and CRP-XL-induced platelet aggregation. To investigate GPVI-induced Ca^2+^ responses, we examined fluorescence changes in response to the same agonists using Fura-2-loaded platelets, employing a 96 well-plate based assay and static conditions [41]. In line with the aggregation results, we did not notice significant changes in [Ca^2+^]_i_ rises by pGRP alone or in combination with pCIB or pCIB^m^ (Figure 8C). These results pointed to near absence of (combined) signaling effects of pGRP and pCIB on platelets under conditions of no or low shear.

## 3. Discussion

In this paper, we unraveled the role of αIIbβ3 integrin-dependent signaling involving PTK2, CIB1, and GPR56 in collagen-dependent thrombus formation under shear conditions. Our data support for this major platelet integrin, the recently recognized concept of integrin affinity and avidity regulation by mechanical forces [21], in that the activation and signaling via αIIbβ3 can be dampened by peptide-mimicking activation of the shear-dependent receptor GPR56. This would imply that external shear forces play a role in the concept of intracellular actomyosin-dependent mechanosensitive forces for αIIbβ3 activation [21]. As we have recently shown [15], the GPIbα-VWF axis contributes to the formation of platelet aggregates under shear.

In our studies, we hypothesized that targeted interference in integrin activation and signaling via PTK2 and CIB1 would affect the process of thrombus formation under shear. To investigate this, we used a range of collagens relevant for the damaged vessel wall, e.g., the fibrillar Horm-type collagen I and human tissue-derived collagens III and IV. These collagen types were previously assigned to a high (I) or lower (III, IV) platelet GPVI-dependency [41]. Earlier work also indicated that the thrombi formed on all three collagens rely on Syk signaling, i.e., on tyrosine kinase activity through GPVI and in synergy with αIIbβ3 signaling [9,41]. As well-characterized PTK2 inhibitors, we used two structurally different compounds, PF573228 and FAK-IN14 [38,39]. We further chemically synthetized the seven-amino acid peptide pGRP, which is considered to mimic shear-dependent self-activation of GPR56 [32] and furthermore a 26-amino acid peptide pCIB (wildtype), known to enter platelets and block the binding of CIB1 to the αIIb chain in outside-in signaling [26], as well as a four-time mutated form pCIB^m^ with calculated higher free binding energy.

Control experiments indicated that pGRP, pCIB or pCIB^m^ by itself did not influence collagen-induced platelet aggregation (integrin inside-out signaling) or Ca^2+^ fluxes, in agreement with a selective action on outside-in signaling. Yet, PTK2 inhibition did block collagen-induced platelet aggregation, pointing to a wider role of PTK2 in GPVI-dependent platelet activation. On the one hand, whole-blood flow experiments indicated that the inhibitors PF573228 and FAK-IN14 alone only slightly affected thrombus formation under high shear, which was also true for pGRP. On the other hand, when pGRP was combined with increasing doses of PF573228 or FAK-IN14, this ultimately annulated the thrombus-forming process, extending over all three collagen preparations and diminishing the majority of parameters. Importantly, the thrombus-suppressing effect of pGRP plus the PTK2 inhibitor was prominent at a high shear rate (1000 s^−1^) and substantially reduced at a low shear rate (150 s^−1^).

Mouse knock-out and human platelet studies have confirmed that PTK2 signaling regulates platelet spreading on fibrinogen [17,37]. Other early studies have shown that PTK2 phosphorylation—and likely activation—is a coordinated signaling event involving integrins as well as other receptors [36,42]. This is in agreement with our current findings that PTK2 inhibition has a larger suppressive effect on GPVI-dependent platelet aggregation than CIB1 peptide interference. However, either type of inhibitor was similarly effective in antagonizing thrombus formation on the investigated collagens. Regarding CIB1, our results are furthermore compatible with the fact that genetic ablation of *Cib1* in mice significantly delayed and destabilized in vivo arterial thrombus formation, while leaving in vitro platelet aggregation unaffected [30].

A remarkable finding was that the combination of the CIB1 peptide (pCIB or pCIB^m^ peptide) with the GPR56 peptide (pGRP) was required to suppress the thrombus-forming process on collagen. Herein, the combination of pGRP + pCIB had the strongest antithrombotic effect, i.e., larger than pGRP + pCIB^m^ or pGRP + PF573228/FAK-IN14. Given the BFE values of pCIB and pCIB^m^, pCIB^m^ was expected to outperform pCIB. However, it appeared that pCIB^m^ was not more effective than its counterpart. This could be due to the fact that pCIB^m^ may adapt a more kinked conformation due to the mutation at Q7, Y11, and Y16, which may induce α-helix conformation of the peptide, resulting in less efficient CIB1-binding. Interestingly, CIB1 has also been reported to bind WASP, a protein with mutations in patients with the immunodeficiency Wiskott-–Aldrich syndrome; the WASP–CIB1 complex was assigned a role in integrin αIIbβ3-dependent cell adhesion [43].

The collective and consistent shear-dependent inhibitory rather than stimulatory effects of pGRP were unexpected. These findings suggest that the tethered ligand-mimicking peptide blocks rather than enhances a positive signaling role of the G13α-linked GPR56 receptor; alternatively, in human platelets, this receptor restricts platelet activation. However, further studies will be needed to confirm this conclusion.

**Figure 9:**
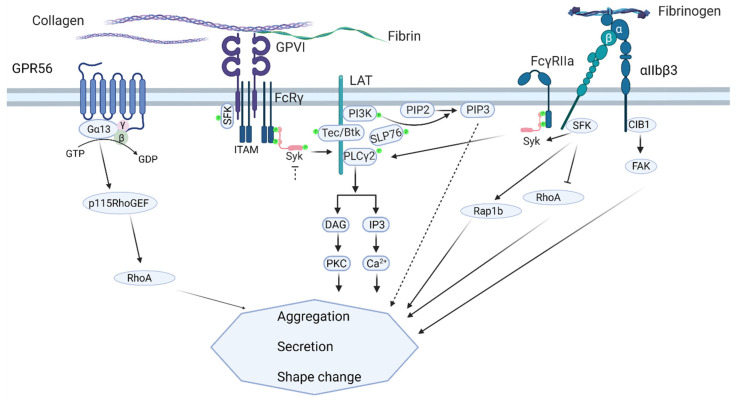
Scheme of the proposed combined action of GPR56, GPVI, and aIIbb3 signaling in shear-dependent thrombus formation on collagen, consisting of platelet aggregation, secretion, and shape change. (i) GPR56 signaling: small GTP-binding protein RhoA, activated by p115 RhoGEF (guanine nucleotide exchange factor). (ii) GPVI signaling via FcR γ-chain co-receptor: SFK (Src-family kinases) and the tyrosine kinases Syk, Tec, and Btk; phosphatidylinositol 3-kinase (PI3K); leading to activation of PLCγ2, which generates the secondary messengers DAG (diacylglycerol) and IP_3_ (inositol trisphosphate). (iii) αIIbβ3 outside-in signaling: SFK- and CIB1-mediated activation, the latter triggering PTK2 (focal adhesion kinase FAK); small GTP-binding proteins Rap1b and RhoA transmit parts of the signal. For further explanation, see text.

Overall, our findings point to a novel shear-dependent role of PTK2 and CIB1 in collagen-induced thrombus formation via integrin activation and signaling that involves the GPR56 receptor (Figure 9). Our work thereby extends the previous studies on the separate roles of PTK2, CIB1, and GPR56. How precisely the presumed GPR56-G13α activity adds to PTK2-CIB1-αIIbβ3 still needs to be disclosed. Supported by earlier knock-out studies [17,27,30,32] and the evaluation of multiple mouse genes in collagen-dependent thrombus formation in vivo and in vitro [14], our findings now add another element to the complex signaling cascades in platelets required for the build-up of a stable contracted thrombus. Our work also extends the multitude of functions of GPR56 in immune regulation and tumor progression [44,45].

## 4. Materials and Methods

### 4.1. Materials

Horm collagen type I derived from equine tendon was obtained from Nycomed (Hoofddorp, The Netherlands). Human placenta-derived collagen type III (1230-01S) was supplied by Southern Biotechnology (Birmingham, AL, USA). Human collagen type IV, FAK inhibitor 14 (FAK-IN14), and PF573228 were obtained from Sigma–Aldrich (Zwijndrecht, The Netherlands). PPACK (D-phenylalanyl-L-propyl-L-arginine chloromethyl ketone) were obtained from Calbiochem (520222, Amsterdam, The Netherlands). Fura-2 acetoxymethyl ester and pluronic were obtained from Invitrogen (Carlsbad, CA, USA). The fluorescent stains used were Alexa Fluor (AF)647-conjugated anti-human CD62P mAb (304918, Biolegend, London, UK), FITC-labeled fibrinogen (F0111, Dako, Amstelveen, The Netherlands), and AF568-labeled annexin A5 (A13202, ThermoFisher, Eindhoven, The Netherlands). Other reagents came from Sigma–Aldrich or from sources described before [41].

### 4.2. Preparation of Blood and Platelets

Blood was drawn from healthy volunteers through venipuncture. Donors had not received anti-platelet medication for at least two weeks and gave full informed consent according to the declaration of Helsinki. Studies were approved by the local Medical Ethics Committee of Maastricht University Medical Centre^+^. Blood from donors was collected into 3.2% trisodium citrate (Vacuette tubes, Greiner Bio-One, Alphen a/d Rijn, The Netherlands). All blood samples had platelet counts within the reference ranges, such as measured by a Sysmex XN-9000 analyzer (Sysmex, Cho-ku, Kobe, Japan).

Platelet-rich plasma (PRP) and washed platelets were isolated, basically as described before [46]. In brief, PRP was obtained from citrated blood by centrifugation at 240 g for 15 min. After the addition of 1:10 *vol*/*vol* acid citrate dextrose (ACD; 80 mM trisodium citrate, 183 mM glucose, 52 mM citric acid), this PRP was centrifuged at 5500 g for 2 min. The pelleted platelets were resuspended in Hepes buffer, pH 6.6 [10 mM Hepes, 136 mM NaCl, 2.7 mM KCl, 2 mM MgCl_2_, 5.5 mM glucose, and 0.1% bovine serum albumin (BSA)]. After the addition of apyrase (1 U/mL) and 1:15 *vol*/*vol* ACD, another centrifugation step was performed to obtain washed platelets. The final platelet pellet was resuspended in Hepes buffer, pH 7.45 (10 mM Hepes, 136 mM NaCl, 2.7 mM KCl, 2 mM MgCl_2_, 5.5 mM glucose, and 0.1% BSA) at the required platelet count as indicated below.

### 4.3. Selection and Design of Peptides

The 7-amino acid peptide TYFAVLM (pGRP), comprising the N-terminal tethered ligand of GPR56, was synthetized as described previously [33]. Other peptides were synthetized to target the interaction site of the EF-hand domain of CIB1 with the integrin αIIb chain in the membrane-proximal hydrophobic 15-amino acid region [47,48]. These included the wildtype CIB1-binding peptide Ace-LVLAMWKVGFFKRNRPP LEEDDEEGQ-OH (pCIB), corresponding to the cytoplasmic C-terminus of the αIIb chain (Leu^983^-Glu^1008^), which has been previously shown to be internalized into platelets [24]. In addition, we designed and synthetized a more hydrophobic, mutated form Ace-LVRKMWQVGFYKRNRYPLEEDDEEGQ-OH (pCIB^m^) and calculated the lowest binding free energy (BFE). For designing the latter, the structure of the αIIb chain was extracted from known NMR analyses (PDB ID: 2KNC), and this was virtually docked onto the binding pocket of CIB1 (PDB ID: 2LM5) by applications of the HADDOCK and HDOCK routines of the protein–protein docking WebServer [49,50]. The retrieved docking poses were refined by performing molecular dynamics simulations, and the BFE values of these docking poses were also calculated and compared in order to determine a likely binding mode of the pCIB–CIB1 complex. The docking solution that gave the lowest BFE, indicative of the most thermodynamically favorable conformation, was chosen as a template for improved in silico design peptide candidates [51,52]. The virtually optimized CIB1–peptide complexes were subjected to molecular dynamics simulations and BFE calculations to predict the most favorable peptide candidate, pCIB^m^, using methods described before [53].

### 4.4. Solid-Phase Synthesis of Peptides

The pCIB peptide was synthesized using automated microwave (CEM Liberty BLUE microwave peptide synthesizer) Fmoc-based synthesis on a Cl-MPA ProTide resin at 0.25 mmol scale. The modified peptide pCIB^m^ was also synthesized at 0.25 mmol scale, but using manual solid-phase peptide synthesis on a methylbenzhydrylamine polystyrene resin (ChemPep, Wellinton, FL, USA), as described previously [54,55]. To produce pCIB^m^, 2-(6-chloro-1H-benzotriazol-1-yl)-1,1,3,3-tetramethylaminium hexafluoro-phosphate (Peptides International, Louisville, KY, USA) was used as coupling reagent. After cleavage of the produced peptide from resin using anhydrous hydrogen fluoride (GHC, Hamburg, Germany), the crude product was analyzed on a Waters (Milford, MA, USA) ultrahigh-performance liquid chromatography mass spectrometric XEVO-G2QToF system. Both peptides were purified by semipreparative HPLC using a Vydac C_18_ HPLC column (10 × 25 mm, 12 mL/min flow rate or 22 × 250 mm, 20 mL/min flow rate; Grace Davison Discovery Sciences, Deerfield, IL, USA) connected to a Waters Deltaprep System consisting of a Prep LC Controller and a 2487 dual wavelength absorbance detector (λ = 214 nm). To elute the peptides, an appropriate gradient of buffer B in buffer A was used, where buffer A was composed of 0.1% trifluoroacetic acid (Biosolve, Valkenswaard, The Netherlands) in H_2_O/CH_3_CN (95/5, *v*/*v*, Biosolve) and buffer B contained 0.1% trifluoroacetic acid in CH_3_CN/H_2_O (90/10, *v*/*v*).

### 4.5. Whole-Blood Thrombus Formation

Microspots of collagen I (M1), collagen III (M2), and collagen IV (M3) were applied by coating degreased coverslips with 0.5 μL of 100 μg/mL, as described previously [10]. After coating, the coverslips were incubated in a humid chamber overnight at 4 °C, washed with saline and blocked with 1% BSA-containing Hepes buffer, pH 7.45, before assembly into the microfluidic chamber [56]. In the case of multiple microspots, the most active one was located downstream to prevent cross-activation of platelets [10].

For the assessment of thrombus formation, samples of 500 µL citrated whole blood were pre-incubated with saline, peptides or inhibitors for 10 min. Immediately before perfusion, blood samples were supplemented with 40 μM PPACK and recalcified with 3.75 mM MgCl_2_ and 7.5 mM CaCl_2_ (f.c.). The recalcified blood was then flowed through a microspot-containing flow chamber for 3.5 min at a wall shear rate of 1000 s^−1^ or 1600 s^−1^ or for 6 min at a wall shear rate of 150 s^−1^. After flow, staining was started by a 2-min perfusion with AF647 anti-CD62P mAb (for P-selectin expression), FITC-fibrinogen (for integrin αIIbβ3 activation), and AF568-annexin A5 (for phosphatidylserine exposure) in Hepes buffer, pH 7.45, containing 2 mM CaCl_2_ and 1 U/mL heparin, as described before [11]. During staining, two brightfield images were captured per microspot. Subsequently, residual label was removed by post-perfusion with Hepes buffer, pH 7.45, containing 2 mM CaCl2 and 1 U/mL heparin, after which three representative multicolor fluorescence images were captured per microspot. All conditions were performed in duplicate runs with blood from at least 3 donors.

### 4.6. Microscopy and Image Analysis

Brightfield and fluorescence images were taken using an EVOS-FL microscope (Life Technologies, Bleiswijk, The Netherlands) equipped with Cy5, RFP, and GFP LEDs, an Olympus UPLSAPO 60x oil-immersion objective, and a sensitive 1360 × 1024 pixel CCD camera [11]. The images were analyzed using semi-automated scripts operating in Fiji (ImageJ) and a scoring procedure based on a preset of reference images [11]. Observers were blinded to the experimental condition. Per microspot, this gave five parameters from brightfield images (P1–5) and one parameter from each of the three-color fluorescence images (P6–8), such as defined in Table 1.

Detailed information on the scripts and scoring procedures is given in the supplementary methods. This section also provides reference brightfield images, used for the scoring. In brief, P1 (platelet adhesion) represents the percentage of total surface-area-coverage (SAC%) occupied by platelets. P2 (platelet aggregate coverage) concerns the SAC% occupied by multilayered platelet aggregates. P3 (thrombus morphological score), P4 (thrombus multilayer score), and P5 (thrombus contraction score) describe the thrombus phenotype, in comparison to refence images, ranging from 0,0,0 (essential absence of platelets) to 5,3,3 (large contracted, multilayered platelet aggregates). Finally, P6 (PS exposure), P7 (P-selectin expression), and P8 (fibrinogen binding) represent %SAC of platelets staining positively for the respective fluorescent probes.

### 4.7. Light Transmission Aggregometry

Aggregation of washed platelets (250 × 10^9^/L) was measured at 37 °C under stirring at 1200 rpm using a chronology aggregometer (Havertown, PA, USA). Platelet samples of 500 µL were pre-incubated with saline, tirofiban (1 μg/mL) or indicated inhibitors (peptides) for 10 min. Platelet activation was performed with indicated agonists at 37 °C.

### 4.8. Cytosolic Ca^2+^ Measurements

Washed platelets (200 × 10^9^/L) were loaded with a mixture of Fura-2 acetoxymethyl ester (3 μM) and pluronic (0.4 µg/mL) in a 40-min incubation at room temperature, as described elsewhere [41]. After centrifugation in the presence of 1:10 ACD and apyrase (1 U/mL), the dye-loaded cells were resuspended at the same concentration into Hepes buffer, pH 7.45. Samples of 200 µL in 96-well plates were pre-incubated with saline, tirofiban (1 μg/mL), or the indicated inhibitor (peptide) for 10 min at room temperature. Subsequently, 1 mM CaCl_2_ was added, and after adaptation to 37 °C, ratiometric changes in fluorescence (excitation wavelengths 340 and 380 nm, emission wavelength 510 nm) were measured per well with a FlexStation 3 (Molecular Devices, San Jose, CA, USA). Agonists collagen I (10 μg/mL, f.c.) or CRP-XL (10 μg/mL, f.c.) were added by roboted pipetting. For optimal, diffusion-limited mixing, the speed of agonist injection was set at 125 μL/s [41]. Calibrated, nanomolar changes in cytosolic [Ca^2+^]_i_ were calculated as before [57,58]. Measurements were performed in triplicate wells, with platelets isolated from at least 3 donors.

### 4.9. Statistics and Data Processing

Statistical analysis was performed with GraphPad Prism 8 software (San Diego, CA, USA). Figures were generated with the same package. Parameter values of thrombus formation from 2–3 corresponding images in the same run were averaged. In addition, parameters of duplicate flow runs were averaged to obtain one parameter set per donor, microspot, and condition [41]. For heatmap representation, mean parameter values across microspots were univariate normalized 0–10 [11]. For statistical analysis, values of control and inhibitor runs were compared per donor, using a paired Student’s *t*-test. In the subtraction heatmaps, a conventional filter was set at *p*-values less than 0.05 [41].

## Figures and Tables

**Table 1:** Overview of obtained parameters (P1–8) of thrombus formation from brightfield and fluorescence images. Measured ranges and scaling factor for heatmap analysis are also indicated. Abbreviations: PS, phosphatidylserine; SAC, surface area coverage. For details of how the parameters were established using scripts in Fiji, see Supplementary Materials.

Parameters		Range	Scaling
*Brightfield images*			
P1	Platelet adhesion (% SAC)	0–71.7	0–10
P2	Platelet aggregate coverage (% SAC)	0–29.8	0–10
P3	Thrombus morphological score	0–4.75	0–10
P4	Thrombus multilayer score	0–2.75	0–10
P5	Thrombus contraction score	0–2.75	0–10
*Fluorescence images*			
P6	PS exposure (% SAC)	0–22.2	0–10
P7	P-selectin expression (% SAC)	0–71.7	0–10
P8	Fibrinogen binding (% SAC)	0–45.7	0–10

## Data Availability

All data are included in the manuscript as figures, tables or supplement figures.

## Supplementary Materials

The following supporting information can be downloaded at: https://www.mdpi.com/article/10.3390/ijms23158688/s1.
