# Supplementary material for: Roles of Focal Adhesion Kinase PTK2 and Integrin αIIbβ3 Signaling in Collagen- and GPVI-Dependent Thrombus Formation under Shear
Source: Int J Mol Sci. 2022 Aug 4;23(15):8688. doi: 10.3390/ijms23158688 (PMC9369275; doi:10.3390/ijms23158688)
Supplement: Supplementary file 1 [file ijms-23-08688-s001.zip › ijms-1810615-supplementary.pdf]

Roles of focal adhesion kinase PTK2 and integrin αIIbβ3 signaling in collagen- and GPVI-dependent thrombus formation under shear

Jingnan Huang,[1-3] Natalie J. Jooss,[1,4] Delia I. Fernández[1,3], Albert Sickmann,[2,5,6] Ángel García,[3] Kanin Wichapong,[1] Ingrid Dijkgraaf[1], Johan W. M. Heemskerk[1,7*]*

This section contains supplementary Figure S1 to Figure S7, and a supplementary methods part.

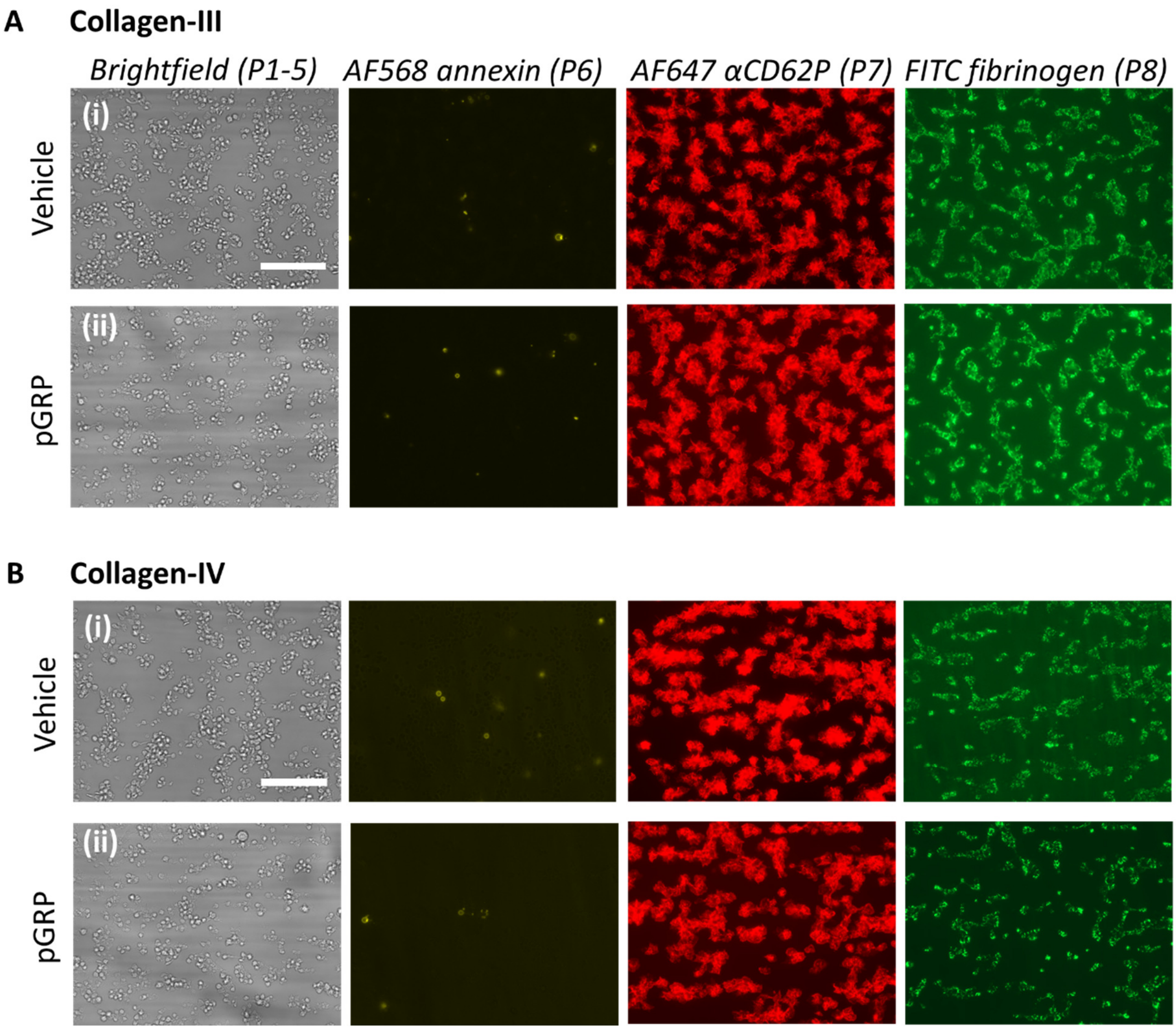

Figure S1. Effects of GPR56-blocking peptide on collagen-dependent thrombus formation. Whole blood (700 µL) was pre-incubated with vehicle medium or pGRP peptide (50 µg/mL) for 10 min. After recalcification, the blood was perfused over microspots of collagen-I, -III and -IV for 3.5 min at wall-shear rate of 1600 s^{-1}. Brightfield and tri-color fluorescence images were taken per microspot at end stage. Shown are representative microscopic images for collagen-III (**A**) and collagen-IV (**B**) of: *(i)* vehicle control, or *(ii)* pGRP. Scale bar = 10 µm.

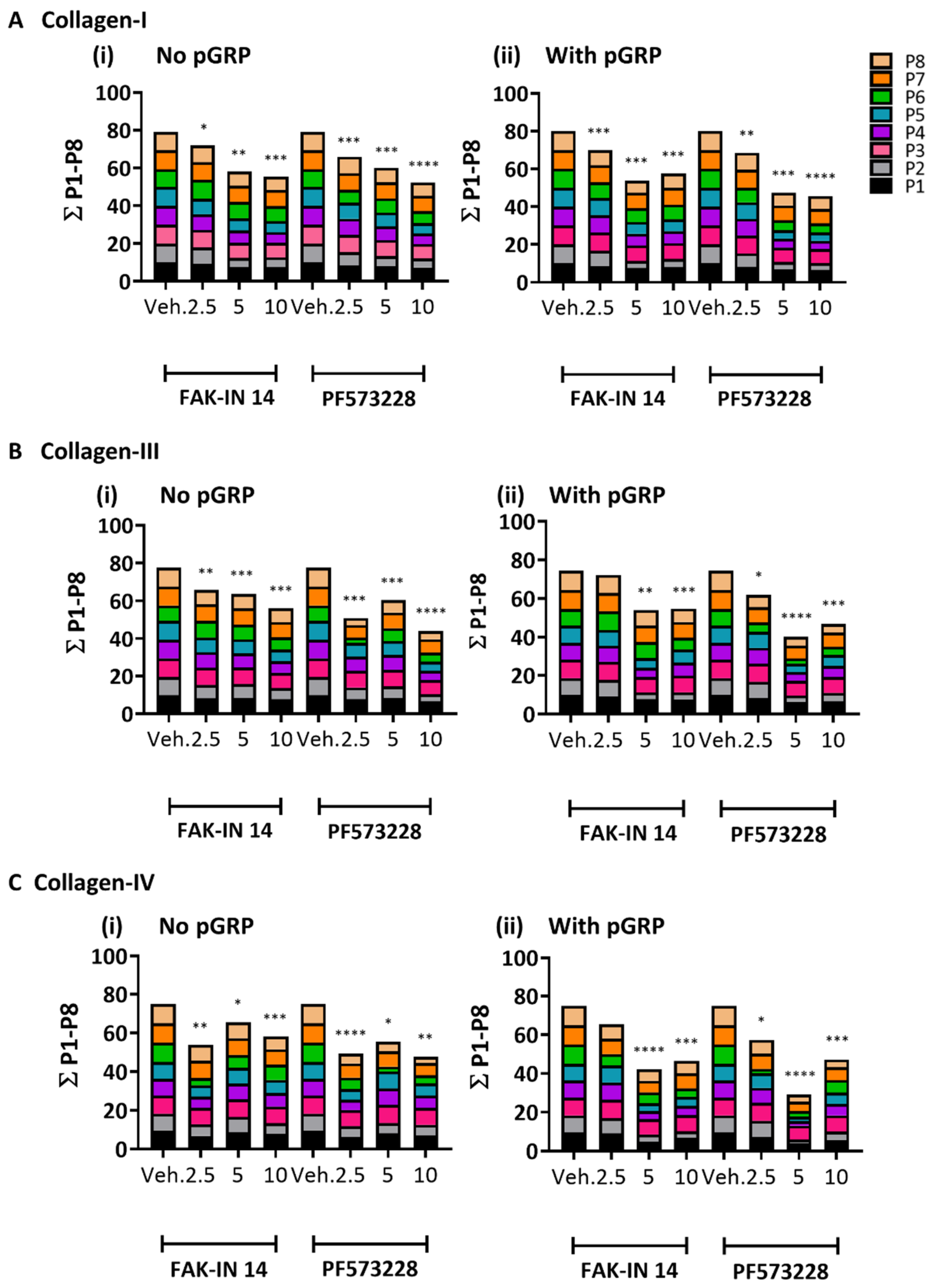

Figure S2. Quantitative effect of GPR56-blocking peptide and PTK2 inhibition on collagen-dependent thrombus formation. Whole-blood flow runs over collagen-I, collagen-III and collagen-VI, and analyzed as for Figure 3. Preincubation of PTK2 was with FAK-IN14 or PF573228 (2.5-10 µM) with(out) pGRP peptide (50 µg/mL). Shown are cumulative plots per condition of scaled (0-10) image parameters: *P1*, platelet adhesion; *P2*, platelet aggregate coverage; *P3-5*, thrombus morphology, multilayer and contraction scores; platelet activation markers: *P6*, PS exposure; *P7*, P-selectin expression; *P8*, fibrinogen binding. Means of duplicate runs for 3-5 donors. Mean values compared per sample using a paired Student's t-test, * $p < 0.05$, ** $p < 0.005$, *** $p < 0.001$, **** $p < 0.0001$.

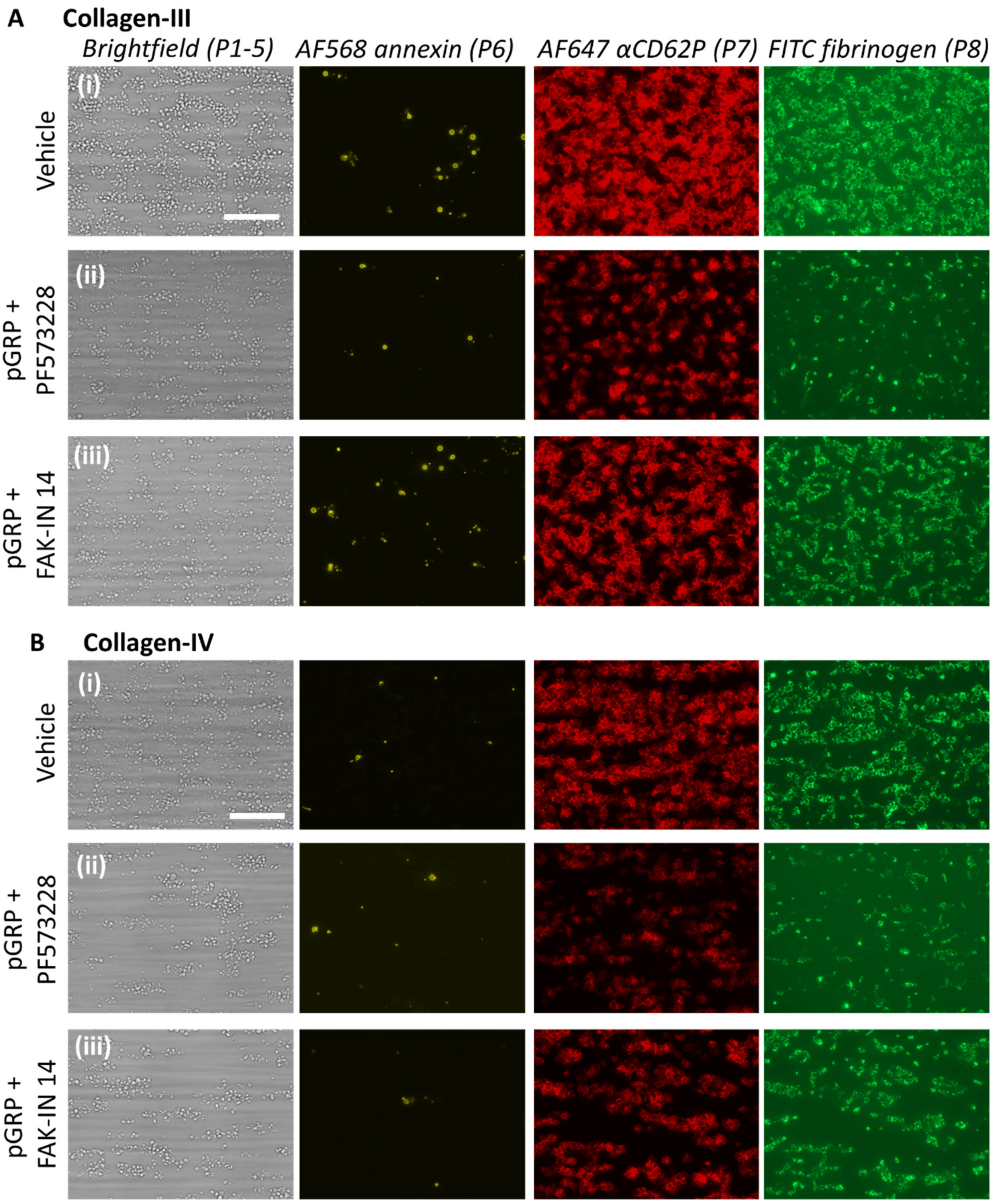

Figure S3. Effect of GPR56-blocking peptide and PTK2 inhibition on collagen-dependent thrombus formation. Whole blood samples were pre-incubated with vehicle medium (control) or indicated PTK2 inhibitor (PF573228 or FAK-IN14, 10 µM) with or without pGRP (50 µg/mL) for 10 min. After recalcification, the blood was perfused over collagen-I, -III and -IV microspots for 3.5 min at standard shear rate of 1000 s^{-1}. Representative end-stage brightfield and tri-color fluorescence images from microspots with collagen-III (**A**) and collagen-IV (**B**). Shown are runs of control *(i)*, pGRP + PF573228 *(iv)* and pGRP + FAK-IN14 *(iii)*. Scale bar = 10 µm.

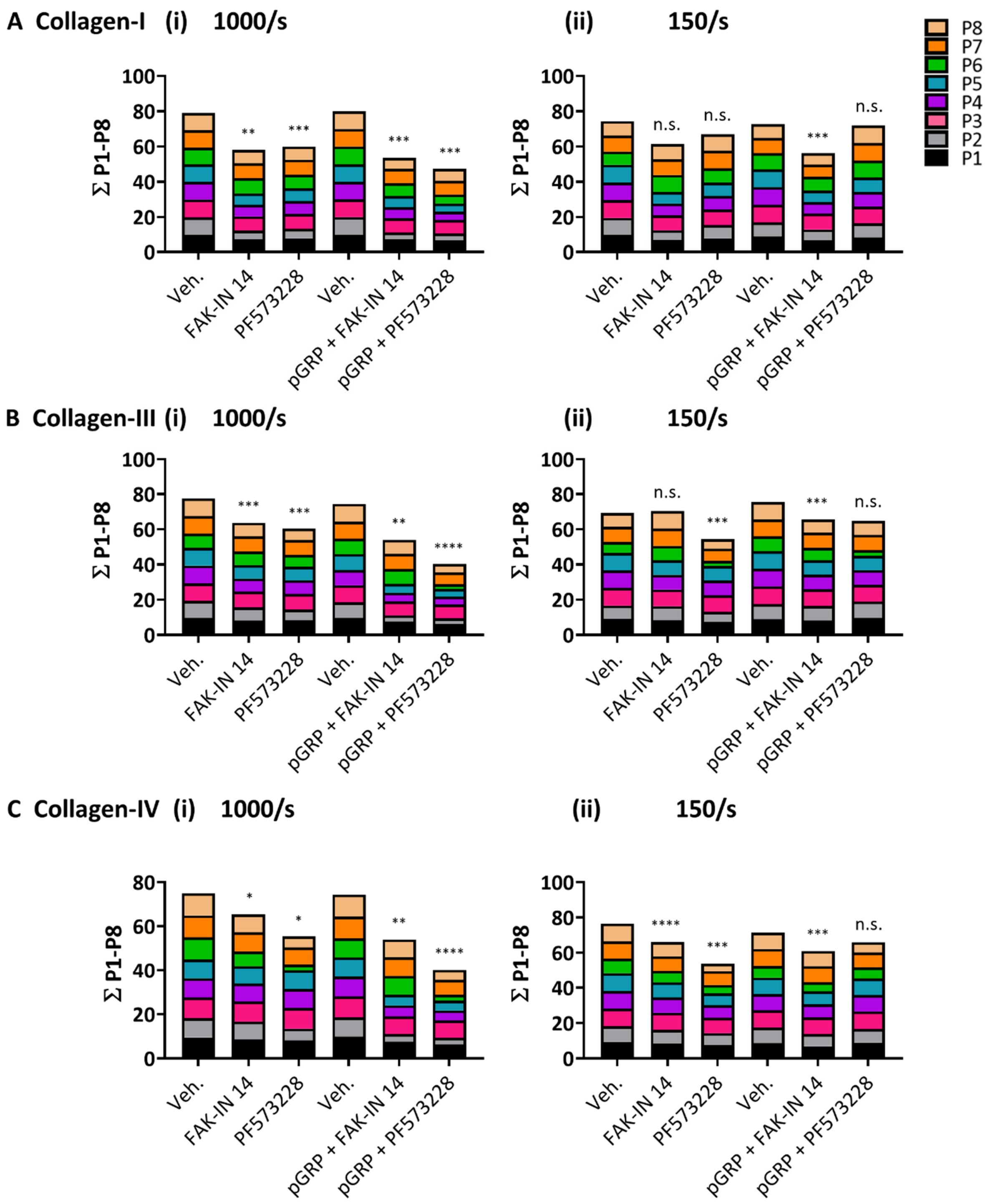

Figure S4. Quantitation of shear rate dependency of GPR56 and PTK2 inhibition. Blood samples pre-incubated with vehicle (control) or PF573228 (5 μM) with/ without pGRP (50 μg/mL) for 10 min, and then perfused over microspots of collagen-I, collagen-III and collagen-IV for 3.5 min at 1000 s^{-1} or for 6 min at 150 s^{-1} (see Figure 4). Shown are cumulative plots per condition of scaled (0-10) parameters *P1-8*. Means of duplicate runs for 3-5 donors. Mean values were compared per each blood sample using a paired Student's t-test, * $p < 0.05$, ** $p < 0.005$, *** $p < 0.001$, **** $p < 0.0001$.

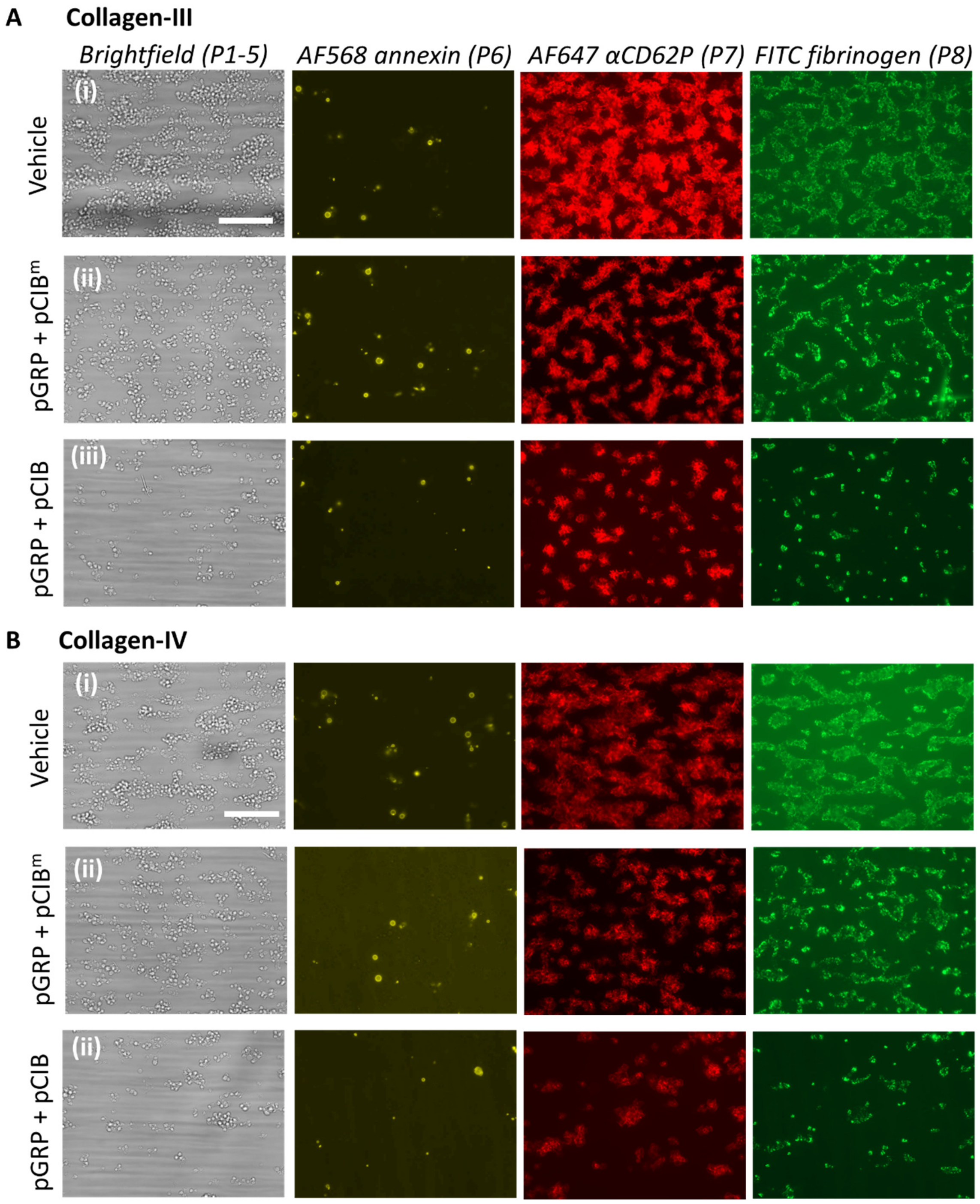

Figure S5. Combined effects of GPR56- and CIB1-blocking peptides on collagen-induced thrombus formation. Whole blood samples were pre-incubated with vehicle (control) or indicated peptides pGRP, pCIB, pCIP^{m} (50 µg/mL each) for 10 min. After recalcification, the blood was perfused over microspots of collagen-I, -III and -IV for 3.5 min at 1000 s^{-1} (as in Figure 6). Brightfield and tri-color fluorescence images were taken per microspot at end stage. Representative images for collagen-III (**A**) and collagen-IV (**B**) of: *(i)* control runs, *(ii)* pGRP + pCIB runs, or *(iii)* pGRP + pCIB^{m} runs. Scale bar = 10 µm. Results from duplicate runs for 3-5 donors.

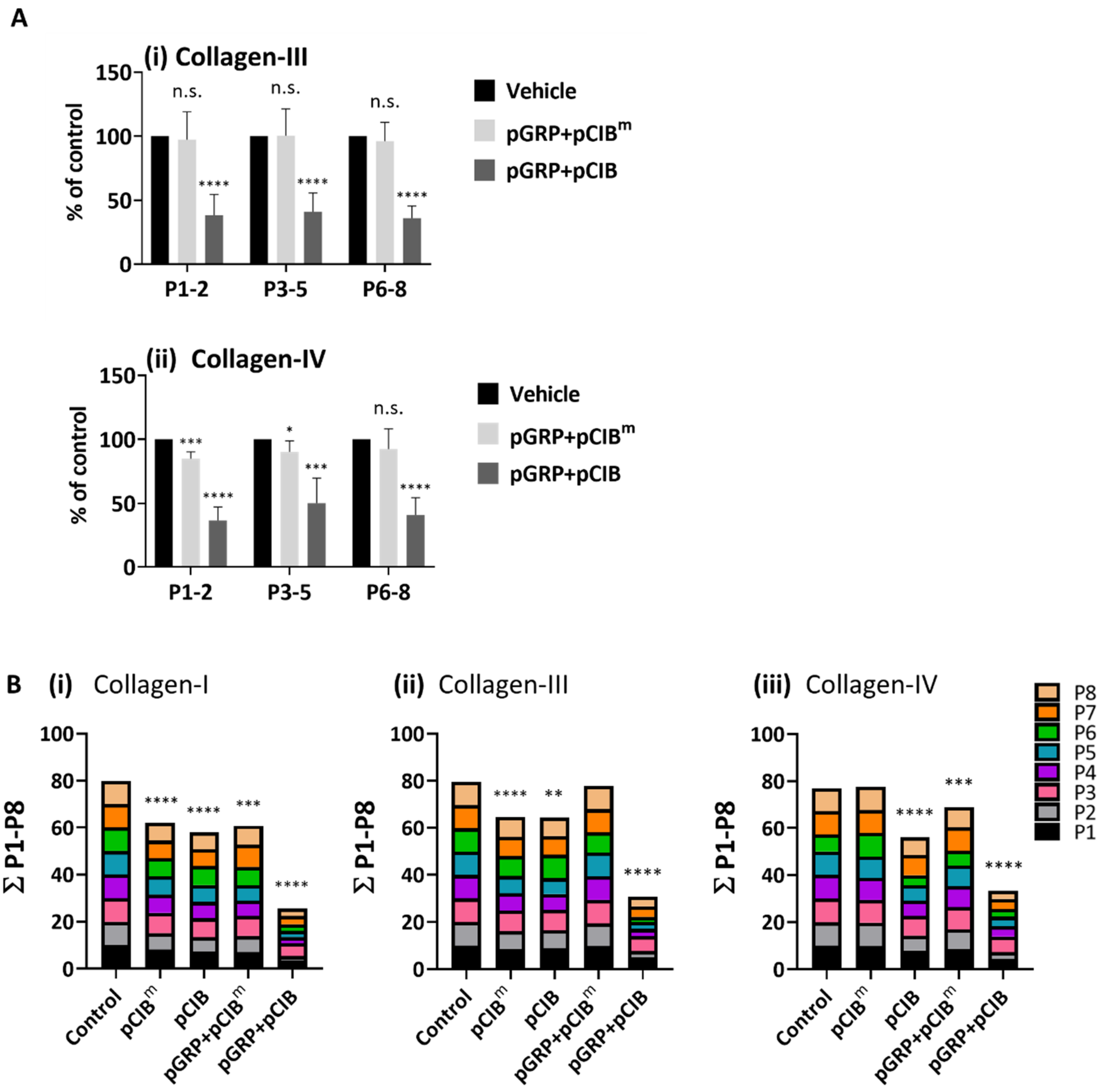

Figure S6. Quantitative effects of GPR56- and CIB1-blocking peptides on collagen-dependent thrombus formation. Experimental setup as in Suppl. Figure 6. (**A**) Percentual effects of peptides on combined parameters of platelet deposition (*P1-2*), thrombus characteristics (*P3-5*) and platelet activation (*P6-8*) versus vehicle control condition for collagen-III (*i*) and collagen-IV (*ii*). (**B**) Cumulative plots per condition of scaled (0-10) parameters *P1-8*. Means of duplicate runs for 3-5 donors. Mean values were compared per each blood sample using a paired Student's t-test, * $p < 0.05$, ** $p < 0.005$, *** $p < 0.001$, ****$p < 0.0001$.

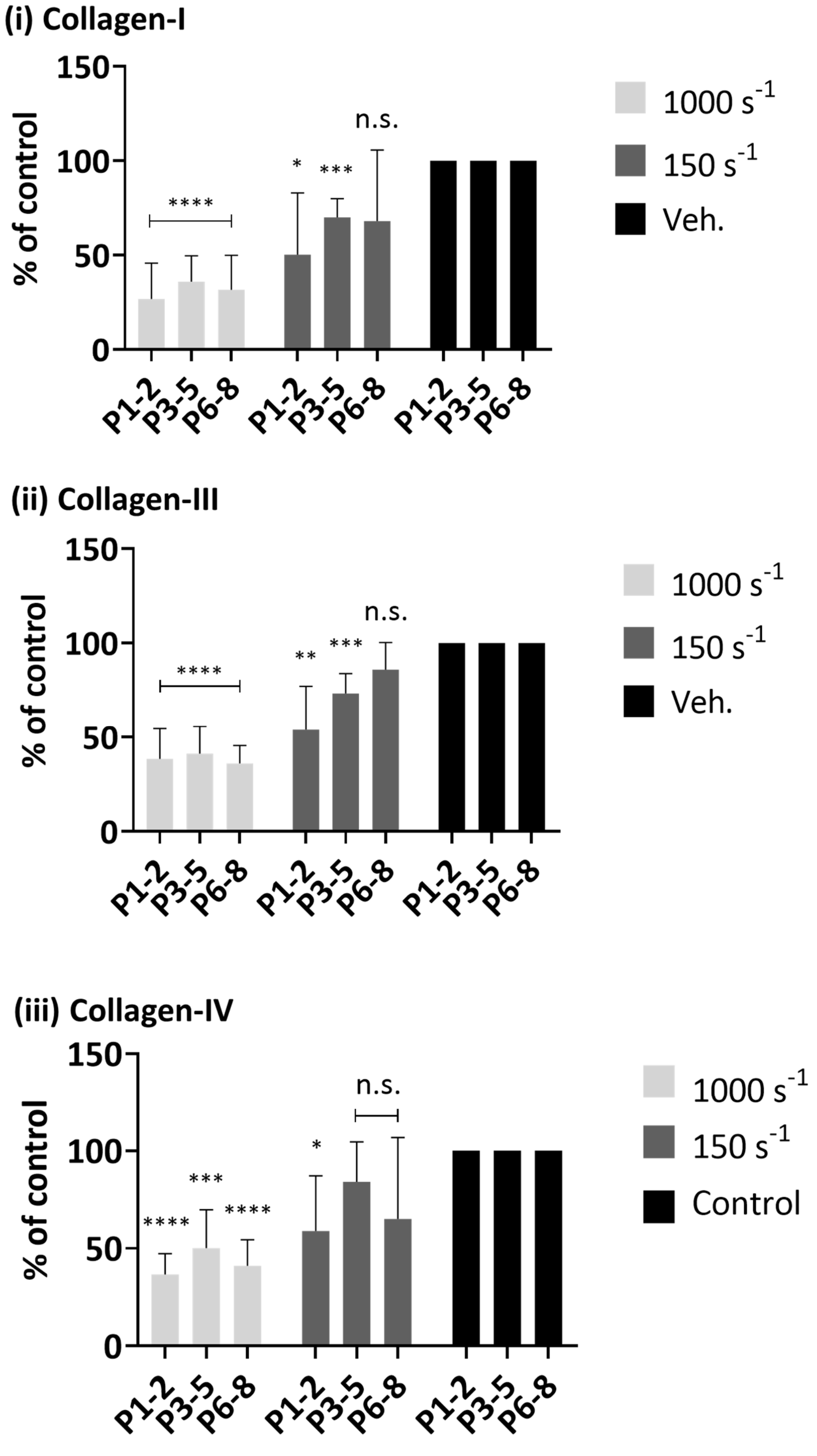

Figure S7. Quantitative effects of rate dependency of GPR56 and CIB1 blockage. Experimental setup as in Figure 7. Blood samples pre-incubated with indicated inhibitors, and perfused over microspots of collagen-I, collagen-III and collagen-IV for 3.5 min at 1000 s^{-1} or for 6 min at 150 s^{-1}. Shown are cumulative plots per condition of scaled (0-10) parameters *P1-8*. Means of duplicate runs for 3-5 donors. Mean values were compared per each blood sample using a paired Student's t-test, * $p < 0.05$, ** $p < 0.005$, *** $p < 0.001$, **** $p < 0.0001$.

Supplementary methods

Image processing for multiparameter assessment of thrombus formation

Microscopic images (EVOS microscope) were separately acquired in brightfield, RPF (probe AF568), CY5 (probe AF647) and GFP (probe FITC) channels, resulting in 8-bit black-white images for brightfield and overlaid 24-bit gray-level fluorescence images in each of the fluorescent channels. All images were analyzed using Fiji software with pre-written scripts for each individual channel. In brief, the scripts opened a set of images using a for-loop. In this loop, the background level was homogenized using a fast Fourier transform bandpass (FFTB) filter. This was followed by manual adjusting a threshold setting and measuring the surface area coverage. For brightfield images, in addition a series of ‘Gray morphology’ conversions was applied to reduce striping and to improve the detection accuracy. The applied conversion steps were as follows: a diamond large-sized close, followed by a medium-sized circle close, and a small circle-shaped dilate. The first step increased the selected pixels, yet stronger in regions with many neighboring pixels; the second step rounded the shapes and additionally reduced straight lines; and the final step could be manually adjusted to match the overlap with the original images and the magnification used in the microscope.

Prior to analysis, the RGB images per color channel were subjected to an FFTB filter with a size to have minimal impact on the structures, and yet flatten the background areas for good analysis. For both the brightfield and CY5 images, large structures were filtered down to 60 pixels; for RFP images large structures were filtered down to 150 pixels; and for GFP images large structures were filtered down to 200 pixels. Small structures were not filtered down as these contained details of interest within platelets.

Reference brightfield images for scoring parameters P3-5

The following microscopic brightfield images were used as a reference for scoring of parameters. Note that scoring was done including halve points.

P3: Thrombus morphological score (range 0-5)
P4: Thrombus multilayer score (range 0-3)
P5: Thrombin contraction score (range 0-3)

Scoring for P3: Thrombus morphology

P3 = 0	P3 = 1	P3 = 2	P3 = 3	P3 = 4	P3 = 5
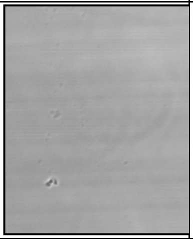	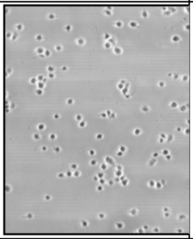	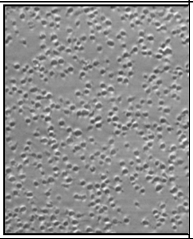	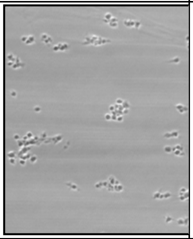	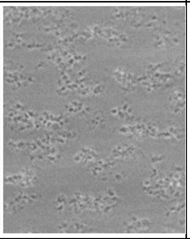	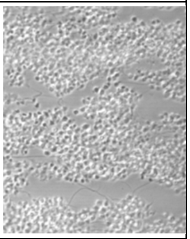
No adhesion	Single platelets	Platelet monolayer	Small aggregates	Medium aggregates	Large aggregates

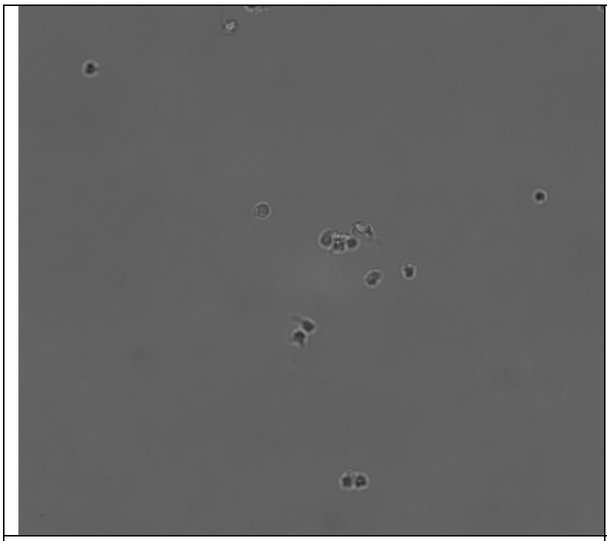	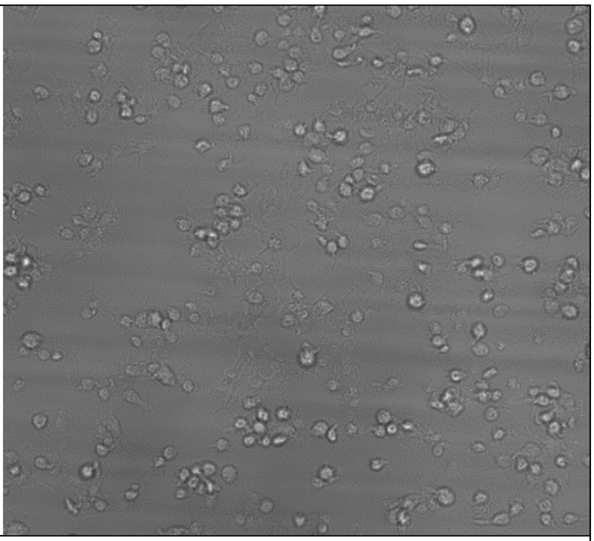
P3: Thrombus morphology 0 (<10 platelets)<br>P4: Thrombus multilayer 0<br>P5: Thrombus contraction 0	P3: Thrombus morphology 1 (>10 platelets)<br>P4: Thrombus multilayer 0<br>P5: Thrombus contraction 0
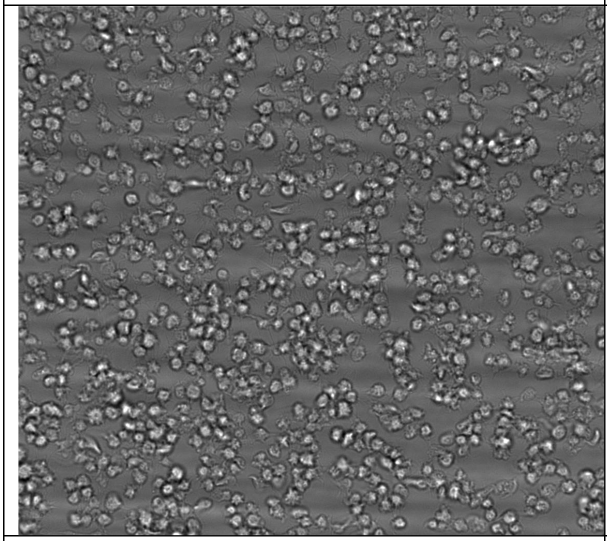	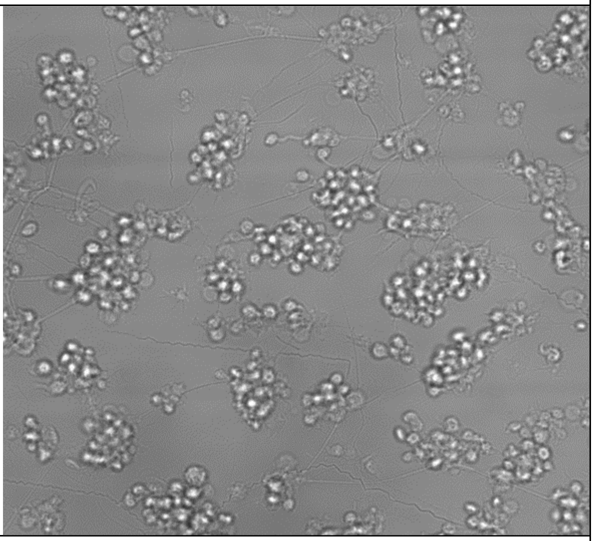
P3: Thrombus morphology 2<br>P4: Thrombus multilayer 0<br>P5: Thrombus contraction 0	P3: Thrombus morphology 3<br>P4: Thrombus multilayer 1<br>P5: Thrombus contraction 1
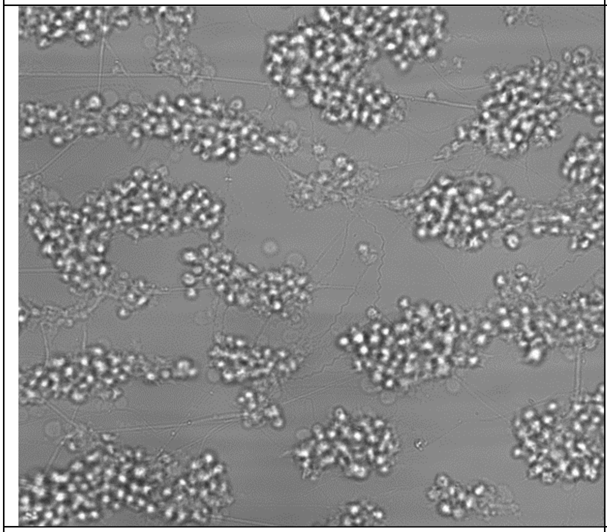	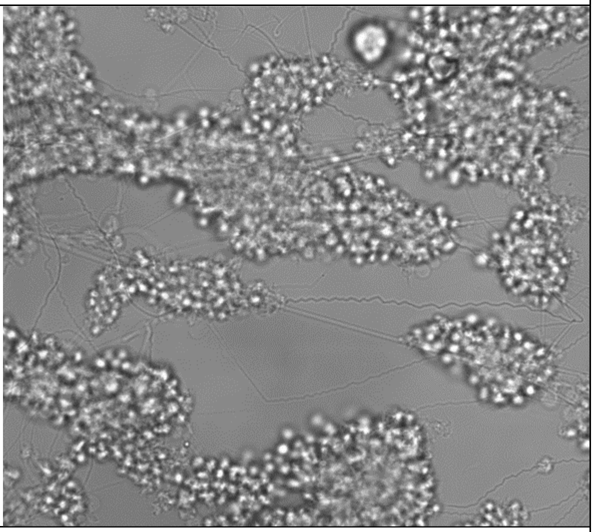
P3: Thrombus morphology 4<br>P4: Thrombus multilayer 2<br>P5: Thrombus contraction 2	P3: Thrombus morphology 5<br>P4: Thrombus multilayer 3<br>P5: Thrombus contraction 3

Performance of script for PS exposure (P6)

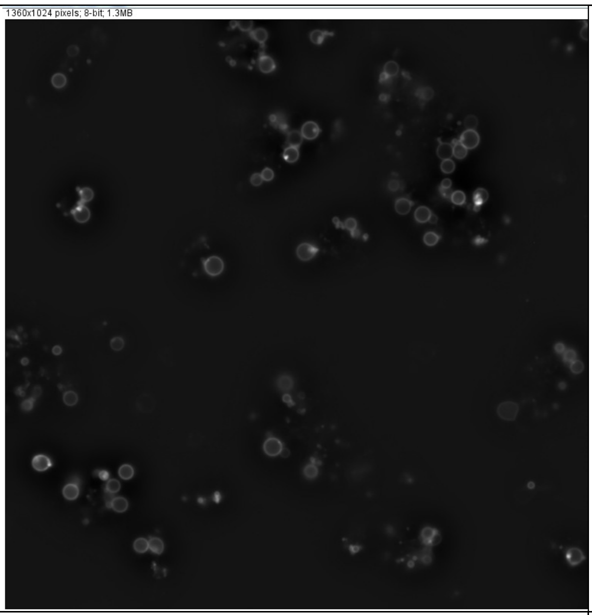

Base image before threshold

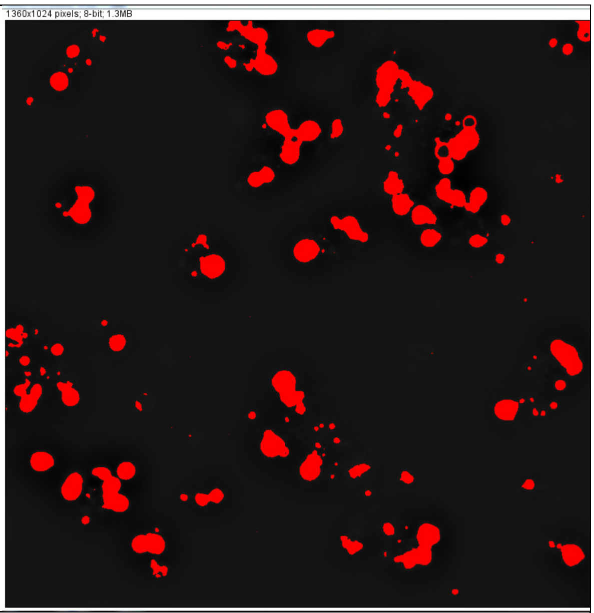

Threshold 25 (optimal) SAC = 6.5%

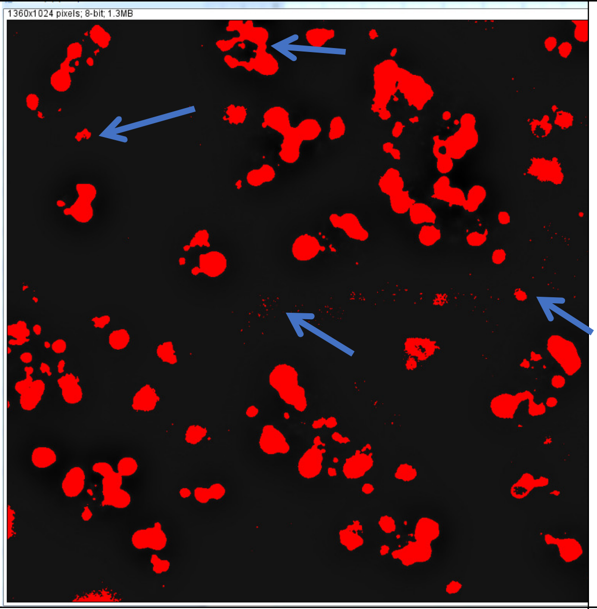

Threshold 21 (too low) SAC = 10.7%

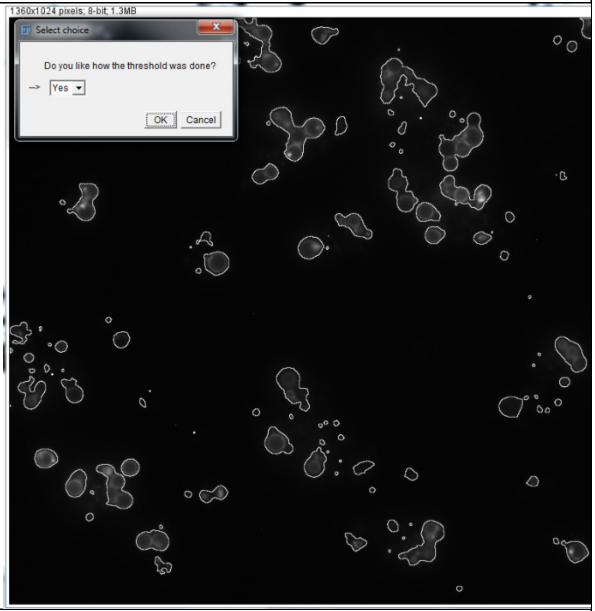

Threshold verification image

Performance of script for P-selectin expression (P7)

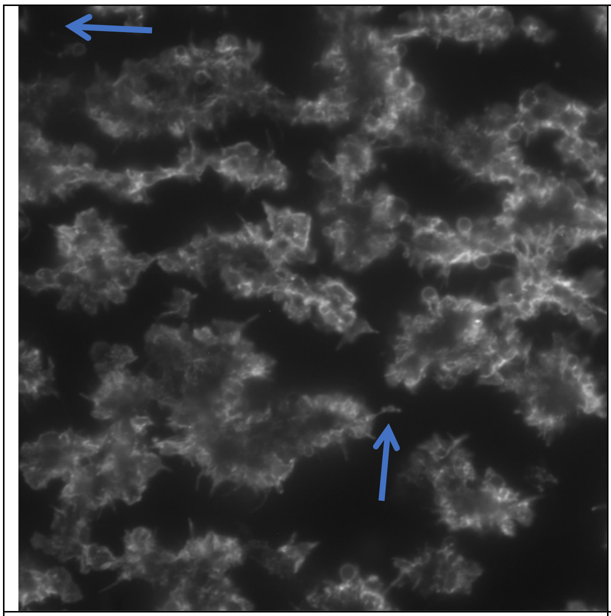

Base image before threshold

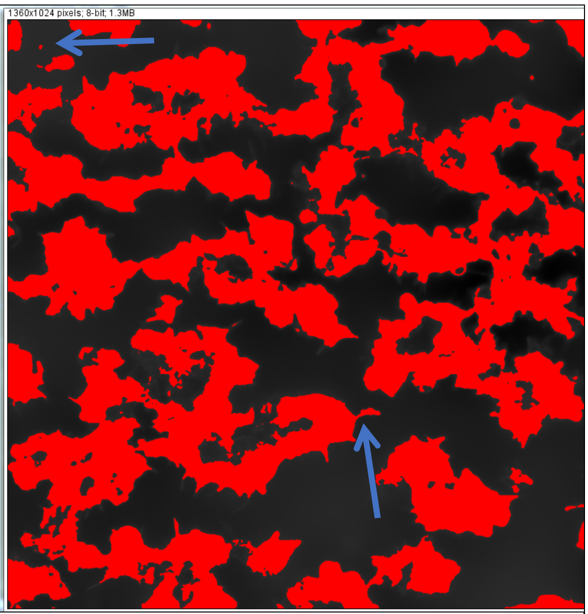

Threshold 45 (optimal) SAC = 44.5%

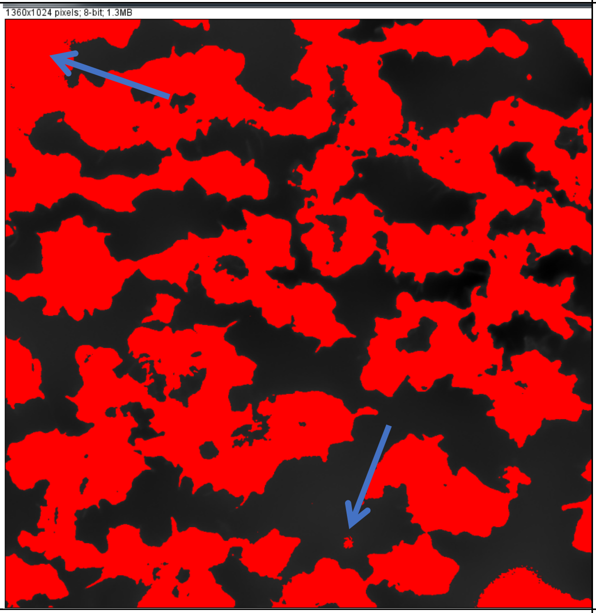

Threshold 40 (too low) SAC = 52.8%

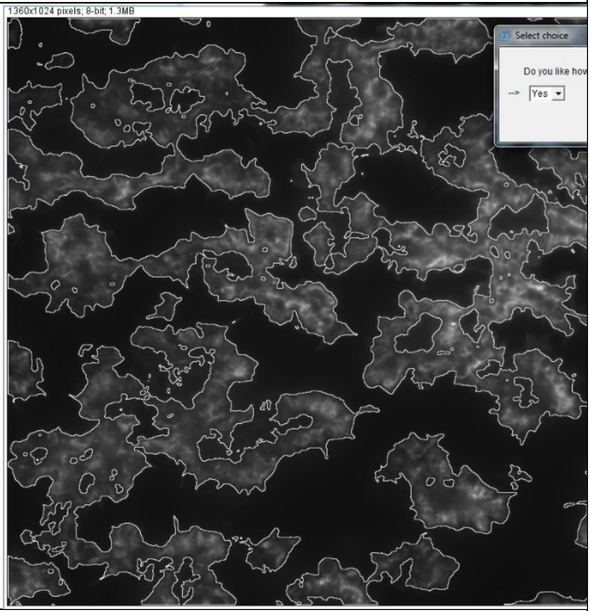

Threshold verification image

Performance of script for fibrinogen binding (P8)

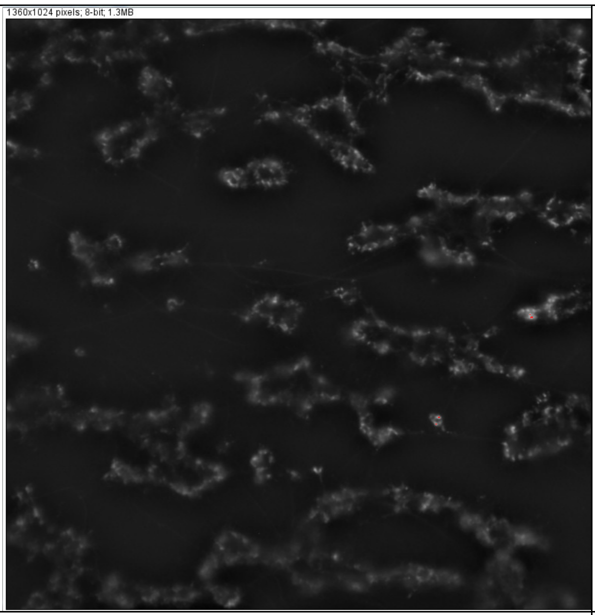

Base image before threshold

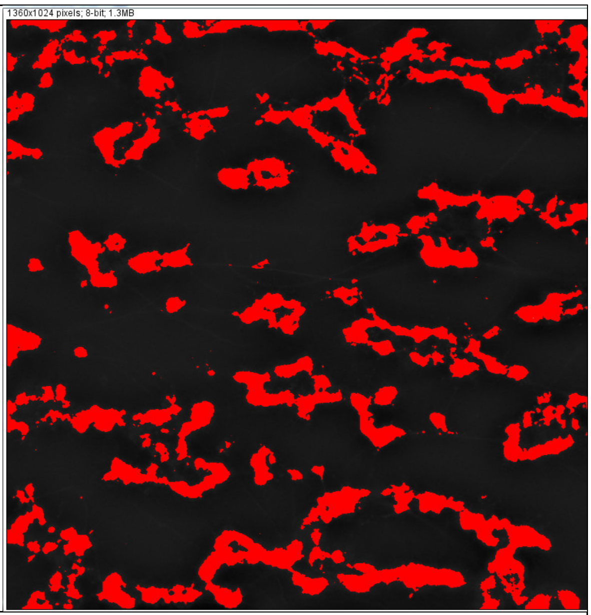

Threshold 33 (optimal) SAC =18.4%

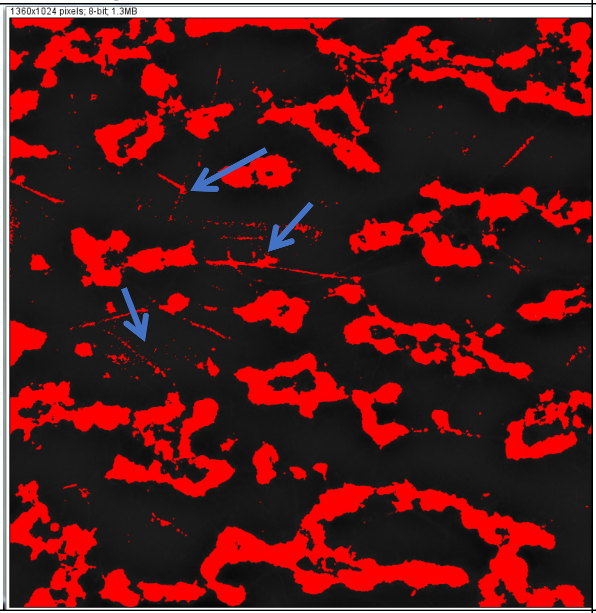

Threshold 29 (too low) SAC= 24.4%

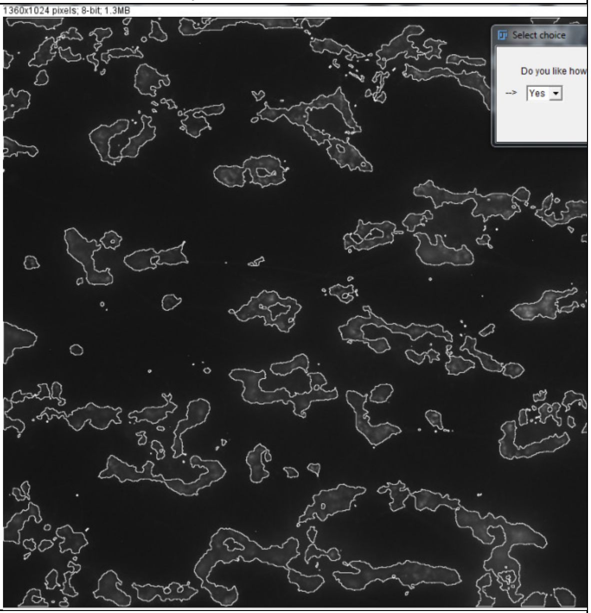

Threshold verification image
